# Thiopurines’ Metabolites and Drug Toxicity: A Meta-Analysis

**DOI:** 10.3390/jcm9072216

**Published:** 2020-07-13

**Authors:** Paula Sousa, Maria Manuela Estevinho, Cláudia Camila Dias, Paula Ministro, Uri Kopylov, Silvio Danese, Laurent Peyrin-Biroulet, Fernando Magro

**Affiliations:** 1 Department of Gastroenterology, Viseu Unit, Tondela-Viseu Hospital Centre, 3504-509 Viseu, Portugal; paula.sousa.6377@hstviseu.min-saude.pt (P.S.); paula.ministro.4342@hstviseu.min-saude.pt (P.M.); 2 Department of Biomedicine, Unit of Pharmacology and Therapeutics, University of Porto, 4200-450 Porto, Portugal; mmestevinho@gmail.com; 3 Department of Gastroenterology, Centro Hospitalar Vila Nova de Gaia/Espinho, 4434-502 Vila Nova de Gaia, Portugal; 4 Department of Community Medicine, Information and Decision in Health, University of Porto, 4200-450 Porto, Portugal; ccamiladias@gmail.com; 5 Centre for Health Technology and Services Research, University of Porto, 4200-450 Porto, Portugal; 6 Department of Gastroenterology, Sheba Medical Central, Ramat Gan and Sackler Medical School, Tel Aviv University, 52621 Ramat Gan, Israel; ukopylov@gmail.com; 7 Department of Biomedical Sciences, Humanitas University, 20090 Milan, Italy; silvio.danese@hunimed.eu; 8 Humanitas Clinical and Research Center, IRCCS, 20089 Milan, Italy; 9 Department of Gastroenterology, Nancy University Hospital, University of Lorraine, 54500 Vandoeuvre-lès-Nancy, France; peyrinbiroulet@gmail.com; 10 Department of Gastroenterology, São João University Hospital, 4200-319 Porto, Portugal; 11 MedInUP, Centre for Drug Discovery and Innovative Medicines, 4200-319 Porto, Portugal

**Keywords:** thiopurines, therapeutic drug monitoring, adverse events

## Abstract

Many questions remain unanswered regarding therapeutic drug monitoring (TDM) utility with thiopurines. This study aims to establish a relationship between thiopurines’ metabolites and drug toxicity. We performed a systematic review with inclusion of studies evaluating the relationship between thiopurines’ metabolites and drug toxicity. Meta-analysis of mean difference (MD), correlations and odds ratio (OR) was performed. We identified 21,240 records, 72 of which were eligible for meta-analysis. Levels of 6-thioguanine nucleotides (6-TGN) were higher in patients with leukopenia (MD 127.06 pmol/8 × 10^8^ RBC) and gastrointestinal intolerance (MD 201.46 pmol/8 × 10^8^ RBC), and lower in patients with hepatotoxicity (MD −40.6 pmol × 10^8^ RBC). We established a significant correlation between 6-TGN and leukocytes (r = −0.21), neutrophils (r = −0.24) and alanine aminotransferase levels (r = −0.24). OR for leukopenia in patients with elevated 6-TGN was 4.63 (95% CI 2.24; 9.57). An optimal cut-off of 135 pmol/8 × 10^8^ RBC for leukopenia was calculated (sensitivity 75.4%; specificity 46.4%). 6-methylmercaptopurine ribonucleotides (6-MMPR) were significantly associated with hepatotoxicity (MD 3241.2 pmol/8 × 10^8^ RBC; OR 4.28; 95% CI 3.20; 5.71). Levels of 6-MMPR measured in the first 8 weeks of treatment were associated with leukopenia. We conclude that TDM could be used to prevent thiopurines’ toxicity. As optimal metabolites level may vary according to indication, physicians may adapt posology to decrease toxicity without compromising efficacy.

## 1. Introduction

Thiopurines (comprising azathioprine (AZA), 6-mercaptopurine (6-MP), and 6-thioguanine) have been used for over 5 decades in the treatment of a myriad of disorders, including acute lymphoblastic leukemia (ALL), inflammatory bowel disease (IBD), auto-immune hepatitis (AIH), and also in the prophylaxis of rejection in organ transplant recipients [1].

As prodrugs, thiopurines have a complex metabolism which leads to the formation of 6-thioguanine nucleotides (6-TGN). Regarding conventional thiopurines, other pathways compete with the production of the active metabolite 6-TGN, leading to the formation of 6-methylmercaptopurine (6-MMP) and 6-MMP ribonucleotides (6-MMPR). These metabolites can be determined by different methods, such as the Lennard [2] and Dervieux–Boulieu assays [3], that perform the measurement in red blood cells (RBC), with concentrations expressed as pmol/8 × 10^8^ RBC.

Thiopurines present toxicity at distinct levels: myelosuppression, hepatotoxicity, pancreatitis and gastrointestinal intolerance, among others. Toxicity is an important cause of treatment cessation; in IBD, about 15% of patients discontinue thiopurines due to adverse events [4,5]. The toxicity of thiopurines can be divided into dose-dependent and idiosyncratic. Due to the distinct metabolisms, the safety profiles of thiopurines may differ. The most worrisome adverse event of 6-thioguanine is liver nodular regenerative hyperplasia (NRH), which still detracts some physicians from its use [6].

The balance between efficacy and toxicity can be achieved with tailored dosing and monitoring, using a weight-based regimen. However, the dose of thiopurines does not correlate with the levels of metabolites [7]. The level of metabolites, specifically 6-TGN, has been associated with improved clinical outcomes in ALL, renal transplantation, and IBD [8,9,10,11]. An optimal therapeutic range of ~230 to 400 pmol/8 × 10^8^ RBC is often cited for patients with IBD and other disorders [12,13]. Values of 6-TGN of 450 pmol/8 × 10^8^ RBC and of 6-MMPR of 5700 pmol/8 × 10^8^ RBC were reported as thresholds for myelotoxicity and hepatotoxicity, respectively [14,15]. However, the benefit of therapeutic drug monitoring (TDM) for thiopurines is still uncertain [16]. Based on the risk of myelosuppression, Food and Drug Administration (FDA) and the Clinical Pharmacogenetics Implementation Consortium recommend genotyping or phenotyping for thiopurine S-methyltransferase (TPMT) deficiency prior to starting thiopurines [17,18,19]. American Gastroenterology Association and proceedings of the first Thiopurine Task Force meeting [6,16] state that the benefit for routine TPMT testing is still uncertain for most patients, and some real-life studies support this statement [20].

Even though this topic is of great interest and can influence the clinical practice in several disciplines, as far as we know, scientific literature lacks a comprehensive study on the metabolites of thiopurines and their correlation with toxicity. The aim of this study was to conduct a systematic review and meta-analysis of the studies associating the levels of thiopurines’ metabolites with the occurrence of toxicity.

## 2. Materials and Methods

### 2.1. Search Strategy

In this study, we followed the Preferred Reporting Items for Systematic Reviews and Meta-Analyses (PRISMA) [21] and the Cochrane Collaboration [22] Guidelines. On November 2018, we performed a literature search on three electronic databases: Pubmed [23], Web of Science [24] and Scopus [25], without time restrictions. The following search words or Medical Subject Heading terms were used: ((“Azathioprine” OR “Mercaptopurine” OR “6-thioguanine nucleotide” OR “6-methyl mercaptopurine”) AND (“Drug-Related Side Effects and Adverse Reactions” OR “adverse effects” OR “leukopenia” OR “toxicity” OR “infection” OR “hepatotoxicity”)). We performed a manual search of the list of references of all relevant studies to ensure that all pertinent articles were considered.

### 2.2. Eligibility and Inclusion/Exclusion Criteria

For our meta-analysis, we considered eligible for inclusion all the studies enrolling adult and/or pediatric patients in which the relationship between thiopurines’ metabolites and toxicity was evaluated. The underlying disease was not a factor for eligibility. The following types of toxicity were considered: myelotoxicity (subdivided in leukopenia, lymphopenia, neutropenia, anemia, and thrombocytopenia), hepatotoxicity, infections, pancreatitis, and/or gastrointestinal intolerance. Oncological adverse events, effects in pregnancy or offspring outcomes, and postsurgical complications were not considered. Randomized controlled trials, cohort studies and case series with more than five patients were considered. No restriction in terms of publication dates was applied. Concomitant medications were allowed but listed.

The exclusion criteria were: (i) systematic reviews or guidelines; (ii) animal studies; (iii) individual case reports; and (iv) case series with up to five patients.

### 2.3. Study Selection and Data Collection

First, we screened the list of titles and the abstracts of the studies identified in the initial search. The list of references was screened by two reviewers and all studies not fulfilling the inclusion criteria were excluded from further analysis. In cases of disagreement, a third independent reviewer was consulted. For the remaining studies, we analyzed full text articles to determine eligibility. Rayyan application (Qatar Computing Research Institute, Doha, Qatar) was used during this process [26].

From the selected studies, we collected the following information: journal and authors’ name, publication year, type of study, cohort’s geographic origin, cohort’s age group (pediatric vs. adult), number of enrolled patients, number of patients with measurement of metabolites, underlying disease for which the thiopurine was used, type of thiopurine and treatment duration, concomitant medication, 6-TGN and 6-MMPR serum levels and cut-offs, methodology used for metabolites quantification, time of metabolites quantification, type of toxicity, definition of toxicity, relationship between thiopurines’ metabolites and drug toxicity.

### 2.4. Quality Assessment

We used funnel plot analysis to detect potential publication bias and/or systematic heterogeneity [22]. The quality of the studies was assessed following the quality assessment tool (QATSDD, Sirriyeh et al, Leeds, UK) [27]. For each study, the scores were added and divided by the maximum possible score (42) to obtain the overall quality score.

### 2.5. Statistical Analysis

In this meta-analysis, the main variable was the occurrence of toxicity. Three types of data were available:

1. Mean values of metabolites concentration in patients with or without toxicity

Since many studies provided medians, in order to avoid losing a significant amount of data by excluding them from the analysis, mean and standard deviation (SD) were calculated from the reported data, as described by Wan et al. (2014) [28]. The studies in which this calculation was performed are identified with an asterisk in the forest-plots. Studies providing full data—allowing the direct calculation of mean and SD—but with a skewed distribution of the variables are identified with two asterisks in the forest-plots. When data were only presented in the form of an image, we extracted the values with WebPlotDigitizer v4.2 (Ankit Rohatgi, Pacifica, CA, USA). Random-effects models were used to test whether mean 6-TGN, 6-MMP or ratio 6-MMPR/TGN values differed among patients with and without toxicity. Review manager v5.3 (Cochrane, London, UK) was used for the evaluation of mean metabolites/ratio differences.

2. Odds Ratio (OR)

When available, the proportion of patients with and without a specific toxicity with levels of 6-TGN/6-MMPR/ratio above and below the defined threshold values was extracted or calculated from each article. However, some studies only provided the final OR value. In these cases, when available, the OR from multivariate analysis was included. Stata 16 (StataCorp, Lakeway Drive, TX, USA) was used for estimating the pooled OR and its 95% confidence interval (95% CI), using a random-effects model. Since different studies used different methods to measure 6-TGN, a previously recommended strategy was used to compare data [29]. The Lennard assay was used as “standard” [2,30,31]; the values of 6-TGN in studies using the Dervieux–Boulieu assay [3] were divided by 2.6 [29], and those obtained with the Erdmann method [32] and with the commercial assay offered by Prometheus Laboratories Inc. (San Diego, CA, USA) were multiplied by 1.6 [33,34]. A high degree of correlation between methodologies has been demonstrated [31,33]. Concentrations of 6-MMPR were not converted, as it has been reported that these are similar in different assays [35,36,37].

3. Correlations

Since some toxicities can be presented as continuous variables, correlation analysis was also performed. The correlation coefficient was extracted or calculated from each article. The Schmidt–Hunter method was used to calculate the overall correlation coefficient (r) from a set of correlations [38]. Statsdirect v3.2.8 (StatsDirect Ltd., Birkenhead, Merseyside, UK) was used for this analysis. 

All the presented *p*-values are two-sided and have a 5% significance level. Statistical heterogeneity was assessed using the I^2^ statistic and by performing subgroup analyses on the following variables: (i) methodology used for the determination of metabolites; (ii) age group; (iii) underlying disease; (iv) geographical origin of the studies; (v) type of thiopurine; (vi) duration of treatment (< or ≥8 weeks); (vii) concomitant medication; and (viii) provided vs. calculated means.

An optimal cut-off for leukopenia was calculated according to the method described by Steinhauser [39].

## 3. Results

### 3.1. Bibliographic Search and Study Selection

**Figure 1:**
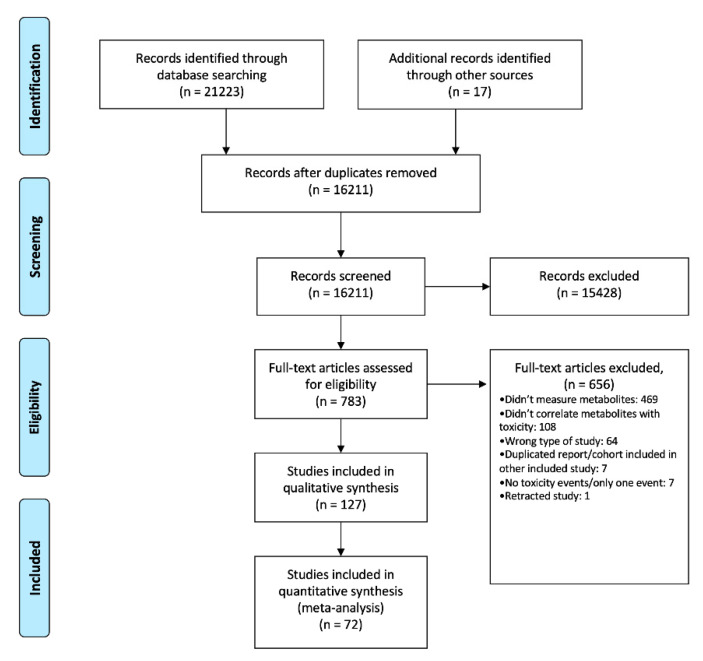
Bibliographic search and study selection—Preferred Reporting Items for Systematic Reviews and Meta-Analyses (PRISMA) flow diagram.

The adopted study selection strategy is detailed in Figure 1. From the initial 21,240 reports, after duplicates removal, initial screening and full-text retrieval and analysis, 127 articles met the criteria for qualitative synthesis. Of these, 72 had appropriate data to be included in the meta-analysis.

### 3.2. Description of the Studies 

The details of the 72 studies included in the meta-analysis are presented in Table 1 and Table S1. The 72 included studies were published between 1983 and 2018. Most studies (n = 60, 83%) were from Western countries. The distribution between pediatric (n = 29, 40%) and adult cohorts (n = 30, 42%) was balanced. Most studies included patients with IBD only (n = 42, 58%), followed by ALL (n = 16, 22%). In the majority of reports, patients were treated with conventional thiopurines (n = 63, 89%) and were being treated for 8 weeks or longer at the time of the first metabolite measurement (n = 35, 49%). Most reports included patients concomitantly treated with other immunosuppressants or chemotherapy drugs. In 31 studies, data of patients without those concomitant drugs were provided and was used in our meta-analysis; still, steroids, mesalamine and/or antibiotics were allowed in most.

The measurement of metabolites followed the procedures described by Lennard [2,30] or were converted to approximate values in 33 studies, the methodology described by Dervieux–Boulieu [3] in 16 studies and the method described by Erdmann et al. [32] in 6 studies. In nine reports the measurements were performed in Prometheus Lab with a proprietary method. The study by Fangbin et al., (2016) [40] used the Dervieux–Boulieu methodology for measurement of 6-TGN and the Lennard method for 6-MMPR. Data for both metabolites (6-TGN and 6-MMPR) were available in 21 studies; 41 studies provided only usable data for 6-TGN and 10 studies for 6-MMPR. In six studies, it was also possible to evaluate the 6-MMPR/6-TGN ratio. We could identify some discrepancies concerning the nomenclature of methylated metabolites. The most common terms were 6-MMPR, 6-MMP, and methylated thioinosine monophosphate (meTIMP). In the methods commonly used for thiopurines’ metabolites measurement, the methylated metabolites are hydrolyzed back to 4-amino-5-methylthiocarbonyl imidazole, a common derivative, rendering them indistinguishable. Therefore, both Dervieux and Lennard assays measure the sum of all methylated metabolites [13,41]. In this paper, the term used to describe these metabolites is 6-MMPR.

Multiple metabolites measurements per-patient were performed in 44 studies, whereas on 17 of them only one determination per patient was performed. When multiple determinations were performed, there was a wide variation concerning the value used in the analysis. When described, the used values are specified in Table 1. The type and definition of toxicities evaluated in each study are detailed in Table S1.

Following Cochrane recommendations, funnel plot asymmetry was only used when at least 10 studies were included [22]; even though in those cases forest plot analysis revealed a considerable amount of variability between studies, funnel plot did not suggest the existence of substantial publication bias.

Regarding quality assessment, the scores ranged from 34.5% [42] to 88.1% [43], with a mean value of 58.9% ± 1.46.

### 3.3. Toxicity and Thiopurines’ Metabolites

#### 3.3.1. Overall Adverse Events

In some reports, the evaluation of toxicity was performed in broader terms, with the inclusion of all toxicities related to thiopurines in the same group.

##### 6-TGN

Ten studies evaluated the differences in 6-TGN levels in patients with and without adverse events [45,46,60,63,69,71,74,79,80,96]. Overall, the mean 6-TGN levels were not different among patients with and without adverse events, with a pooled difference of 12.41 pmol/8 × 10^8^ RBC (95% CI, −76.18; 51.35; *p* = 0.70). We could notice a significant heterogeneity among these studies (*p* < 0.01; I^2^ = 80%). In subgroup analysis, age group was partially responsible for studies’ inconsistency (*p* = 0.04; I^2^ = 69%). Studies including only adult patients had significantly higher levels of 6-TGN in patients with adverse events (mean difference of 77.62 pmol/8 × 10^8^ RBC, 95% CI 38.39; 116.84; *p* < 0.01) (*p* = 0.66; I^2^ = 0%) (Figure S1). The use of concomitant medication was not a significant factor accounting for the observed heterogeneity (*p* = 0.84; I^2^ = 0%). When the analysis was restricted to studies without concomitant medication (such as chemotherapeutic agents or other immunosuppressors), the mean 6-TGN levels were not different among patients with and without adverse events (mean difference of 23.79 pmol/8 × 10^8^ RBC, 95% CI −135.21; 182.80; *p* = 0.77) (*p* < 0.01; I^2^ = 82%).

Four studies provided data to calculate a pooled OR [46,71,79,96] for thresholds of 136.5, 384, 400 and 400 pmol/8 × 10^8^ RBC. Patients with 6-TGN levels above the predefined thresholds were almost three times more likely to have adverse events (OR = 2.58, 95% CI 1.36; 4.90; *p* < 0.01) (*p* = 0.33; I^2^ = 12%). When the analysis was restricted to studies using thiopurines in monotherapy, patients with 6-TGN levels above the predefined thresholds were more than three times more likely to experience adverse events (OR 3.52, 95% CI 1.84; 6.75; *p* < 0.01) (*p* = 0.95; I^2^ = 0%).

##### 6-MMPR

Mean levels of 6-MMPR were significantly higher in patients with adverse events, with a pooled difference of 1184.82 pmol/8 × 10^8^ RBC (95% CI 147.00; 2222.64; *p* = 0.03) (Figure S2) [46,60,74,79,80]. The existing heterogeneity (*p* < 0.01; I^2^ = 90%) was reduced (*p* = 0.05; I^2^ = 62%) when the study by Cuffari et al., 1996 [60] was excluded from the analysis. With the exclusion of this study, the levels of 6-MMPR were similar in patients with and without adverse events. Subgroup analysis did not clarify the origins of inconsistency. Similarly, 6-MMP levels were not different in patients with and without adverse events in those studies using thiopurines in monotherapy, but with considerable heterogeneity between studies (mean difference of 3253.57 pmol/8 × 10^8^ RBC, 95% CI −2547.48; 9054.62; *p* = 0.27) (*p* < 0.01; I^2^ = 96%).

#### 3.3.2. Myelotoxicity

##### “General” Myelotoxicity

In some reports, toxicity combined different aspects of bone marrow suppression, here represented as “general” myelotoxicity.

##### 6-TGN

In the pooled analysis of four studies, 6-TGN levels in patients with myelotoxicity showed no differences [46,48,58,80], with a mean difference of 169.14 pmol/8 × 10^8^ RBC (95% CI −69.09; 407.37; *p* = 0.16). However, the heterogeneity was high (*p* < 0.01; I^2^ = 93%). In the sub-analysis by duration of thiopurine treatment, only studies including patients with <8 weeks of therapy when metabolites were assessed showed numerically higher mean 6-TGN levels in patients with myelotoxicity (Figure S3). A single study reported including patients using thiopurines in monotherapy [48]; in this study there were no differences in 6-TGN levels in patients with and without myelotoxicity, but only patients with more than 3 months of treatment were included.

Three studies provided data to calculate a pooled OR, all of them using a threshold of 450 pmol/8 × 10^8^ RBC [36,46,58]. The risk of myelotoxicity was almost eight times higher in patients with elevated 6-TGN levels (OR = 7.78, 95% CI 1.67; 36.34; *p* < 0.01) (*p* = 0.24; I^2^ = 31%). In one of these studies, thiopurines was used as part of a chemotherapy regimen, while in the other two concomitant medications were not specified.

##### 6-MMPR

In the pooled analysis of four studies, 6-MMPR levels in patients with myelotoxicity were not significantly different [46,58,79,80], with a mean difference of 1601.12 pmol/8 × 10^8^ RBC (95% CI −559.56; 3761.79; *p* = 0.15) (*p* = 0.08; I^2^ = 55%). When the analysis was restricted to studies with IBD patients, 6-MMPR levels were significantly higher in patients with myelotoxicity, with a mean difference of 3529.87 pmol/8 × 10^8^ RBC (95% CI 295.65; 6764.09; *p* = 0.03).

Three studies provided data to calculate a pooled OR; two of them used a threshold of 5700 pmol/8 × 10^8^ RBC [35,58] and the other used a threshold of 11450 pmol/8 × 10^8^ RBC [80]. There was no significant association between 6-MMPR concentrations and overall myelotoxicity (OR 3.83; 95% CI 0.47; 31.37; *p* = 0.21) (*p* = 0.03, I^2^ = 70%).

##### 6-MMPR/6-TGN Ratio

The 6-MMPR/6-TGN ratio was not significantly different in patients with and without myelotoxicity (mean difference 115.54, 95% CI −109.31; 340.40; *p* = 0.31), in the pooled analysis of two studies [46,58], but with considerable heterogeneity (*p* < 0.01, I^2^ = 100%).

##### Anemia

6-TGN

**Figure 2:**
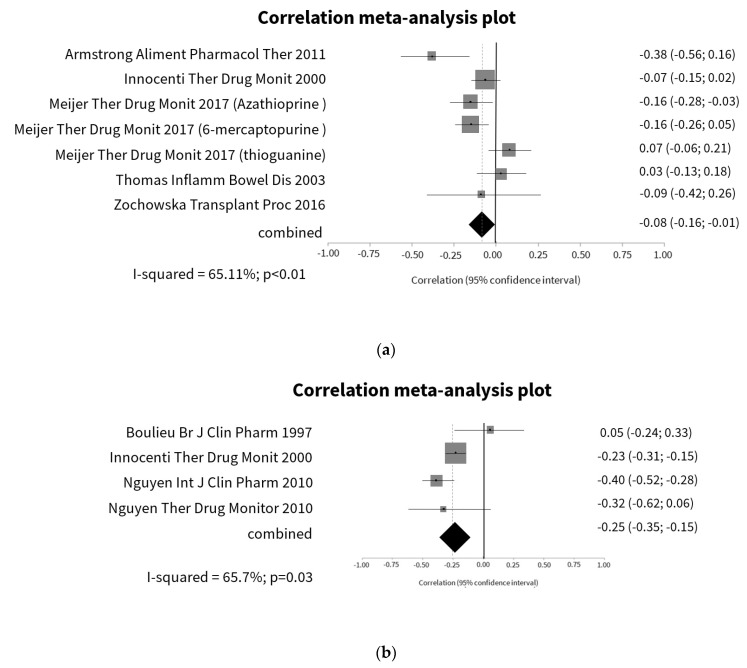
Correlation meta-analysis plot of 6-thioguanine nucleotides (6-TGN) and anemia. (**a**) Relationship between 6-TGN and hemoglobin; (**b**) relationship between 6-TGN and erythrocytes count.

On this topic, 5 studies [34,36,48,81,108] evaluated the relation between 6-TGN levels and hemoglobin and registered a significant but weak weighted mean correlation (Figure 2a). When the analysis was restricted to studies with IBD patients [34,36,48], the strength of the correlation improved (r = −0.28, 95% CI −0.50; −0.06; *p* = 0.01) (Figure S4). However, in the two studies that included patients on thiopurines monotherapy, the weighted mean correlation was not statistically significant, albeit with considerable heterogeneity (r = −0.10, 95% CI −0.36; 0.17; *p* = 0.48) (*p* < 0.01, I^2^ = 88%). Regarding the relationship between 6-TGN levels and erythrocytes count [54,81,93,94], the heterogeneity between studies was substantial and the correlation between variables was significant but weak (Figure 2b). The strength of the correlation improved when the analysis was restricted to studies with patients on thiopurines monotherapy (r = −0.39, 95% CI −0.42; −0.35; *p* < 0.01), with no heterogeneity among studies (*p* = 0.01, I^2^ = 0%).

##### Leukopenia

6-TGN

**Figure 3:**
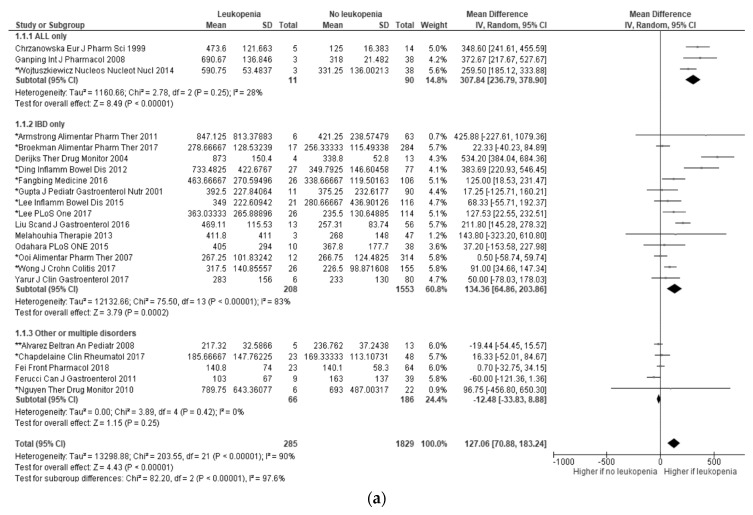

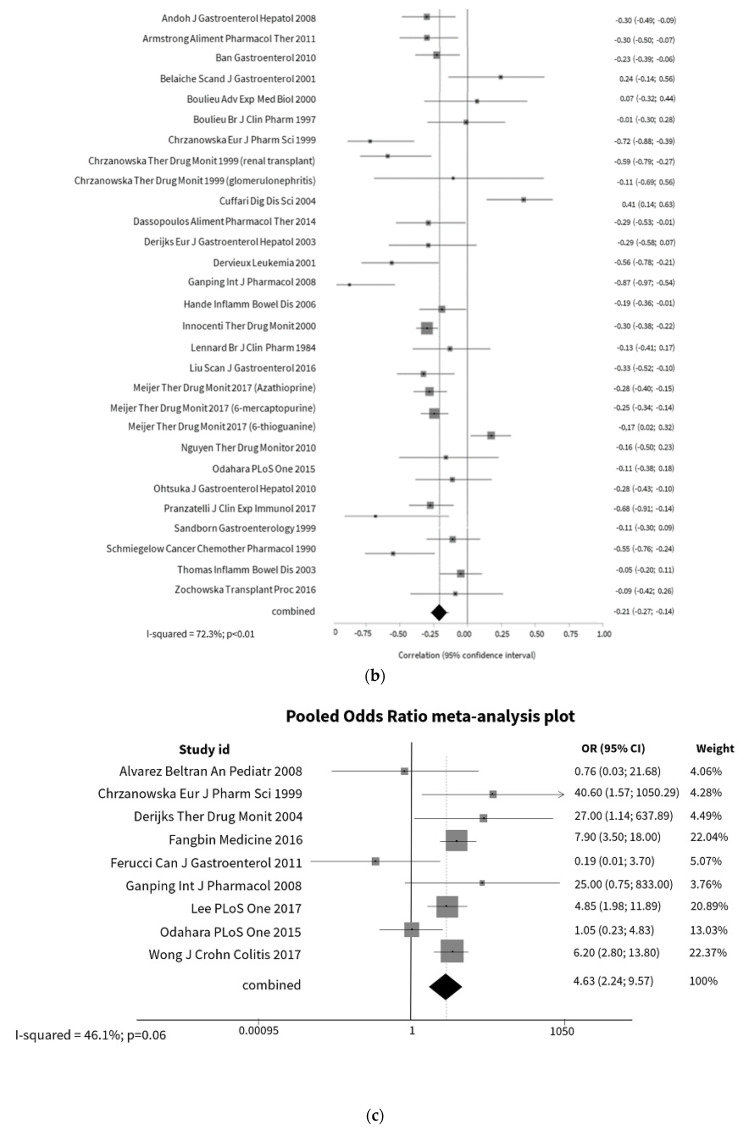
Relationship between 6-TGN levels and leukopenia. (**a**) Means difference forest plot with sub-analysis by disorder (* studies in which mean was calculated from medians; ** studies in which mean was calculated from a sample with skewed distribution; ALL: acute lymphoid leukemia; IBD: inflammatory bowel disease); (**b**) correlation meta–analysis plot; (**c**) odds ratio meta-analysis plot.

In a pooled analysis of 22 reports [40,46,48,56,57,58,65,67,70,72,73,75,84,85,91,92,94,96,98,103,105,107], 6-TGN concentrations were significantly higher in patients with leukopenia (mean difference of 127.06 pmol/8 × 10^8^ RBC (95% CI 70.88; 183.24; *p* < 0.01)). We could observe a considerable heterogeneity between studies (*p* < 0.01; I^2^ = 90%). The underlying disease showed to have influence on the results: 6-TGN levels were higher in patients with leukopenia only in leukemic and IBD patients, whereas no differences could be reported for other disorders (Figure 3a). Restricting the analysis to patients using thiopurines in monotherapy did not change the results (mean difference of 120.44 pmol/8 × 10^8^ RBC (95% CI 31.06; 209.83; *p* < 0.01) (*p* < 0.01; I^2^ = 86%).

Twenty-six studies showed a negative correlation between 6-TGN and leukocytes, with a weak weighted mean (Figure 3b) [34,36,43,47,48,49,51,54,55,58,59,61,62,64,66,73,77,81,87,91,94,96,97,99,101,108]. Similar results were obtained when the analysis was limited to patients with thiopurines in monotherapy (r = −0.18, 95% CI −0.25; −0.12; *p* < 0.01) (*p* = 0.30; I^2^ = 15%). The strength of correlation was stronger when the analysis was restricted to studies with ALL patients (r = −0.35, 95% CI −0.46; −0.24; *p* < 0.01) [58,66,73,81,101], pediatric cohorts (r = −0.33, 95% CI −0.41; −0.25; *p* < 0.01), and patients with < 8 weeks of treatment at the time of the first metabolite assessment (r = −0.31, 95% CI −0.43; −0.20; *p* < 0.01). However, a substantial heterogeneity between studies was noticed.

Nine studies provided data to calculate a pooled OR [40,46,58,65,72,73,85,96,105], with thresholds ranging from 127 to 450 pmol/8 × 10^8^ RBC. In these studies, patients with high 6-TGN levels were more than four times more likely to have leukopenia than those with lower levels (Figure 3c). In the pooled analysis of the three studies with thiopurines in monotherapy, patients with high 6-TGN levels were almost six times more likely to have leukopenia than those with lower levels (OR 5.87, 95% CI 3.27; 10.55; *p* < 0.01) (*p* = 0.58; I^2^ = 0%).

Noticeably, in the sub-analysis by leukopenia definition, only studies defining leukopenia as a white blood count below 3 or 3.5 × 109/L had a significant association both in mean difference (MD) and OR analysis.

The data provided for different cut-offs enabled us to calculate an optimal cut-off of 135 pmol/8 × 10^8^ RBC for leukopenia, with a sensitivity of 75.4% and specificity of 46.4% (area under the curve (AUC) = 0.629, 95% CI 0.432; 0.837) (Table S2).

6-MMPR

The analysis of a pool of six studies demonstrated that the levels of 6-MMPR were not significantly higher in patients with leukopenia [46,58,72,85,92,105] (mean difference of 277.00 pmol/8 × 10^8^ RBC (95% CI −237.06; 791.07; *p* = 0.29), with similar results when the analysis was limited to patients medicated with thiopurines in monotherapy (mean difference of 1161.78 pmol/8 × 10^8^ RBC (95% CI −934.44; 3258.01; *p* = 0.28) The heterogeneity between studies was substantial (*p* < 0.01 for both; I^2^ = 67% and 84%, respectively). In the subgroup analysis, method and time of treatment were significant variables. In fact, only studies using the Lennard method for metabolites assessment, and studies including patients with less than 8 weeks of treatment at the time of measurement reported higher levels of 6-MMPR with leukopenia (Figure S5).

Our analysis could not denote a significant correlation between 6-MMPR levels and leucocytes (r = −0.04, 95% CI −0.12; 0.04; *p* = 0.29) (Figure S6) [36,37,55,58,94,108]. However, when the analysis was restricted to studies including patients with less than 8 weeks of treatment at the time of assessment, the correlation was significant (r = −0.22, 95% CI −0.34; −0.09; *p* < 0.01).

The pooled analysis of three studies that provided data for OR calculation revealed that there was no association between 6-MMPR levels and leukopenia (OR 2.02, 95% CI 0.37; 10.90; *p* = 0.42) (*p* = 0.09; I^2^ = 59%). However, in the one study that only included patients with less than 8 weeks of treatment at the time of assessment [105], patients with 6-MMPR levels above 3525 pmol/8 × 10^8^ RBC were almost six times more likely to develop leukopenia (OR 5.9, 95% CI 2.7–13.3) [46,58,105]. Additionally, of the three studies, this was the only study reporting the use of thiopurines in monotherapy.

6-MMPR/6-TGN Ratio

We could evidence that the 6-MMPR/6-TGN ratio was not significantly different in patients with leukopenia [46,56,58,105] (Figure S7). Two studies correlated this ratio with leukocytes [55,58], with a weighted mean correlation of 0.31 (95% CI 0.15; 0.46, *p* < 0.01) (*p* = 0.44; I^2^ = 0%).

##### Neutropenia

6-TGN

The mean levels of 6-TGN were similar in neutropenic and non–neutropenic patients, but there was a considerable degree of heterogeneity (MD 249.01 pmol/8 × 10^8^ RBC; 95% CI −276.97; 774.99; *p* = 0.35) (*p* < 0.01; I^2^ = 98%) [58,84]. Two studies calculated the mean level of neutrophils above and below a threshold of 200 and 210 pmol/8 × 10^8^ RBC of 6-TGN [52,86]. The levels of neutrophils were significantly higher in the low 6-TGN group (Figure S8).

**Figure 4:**
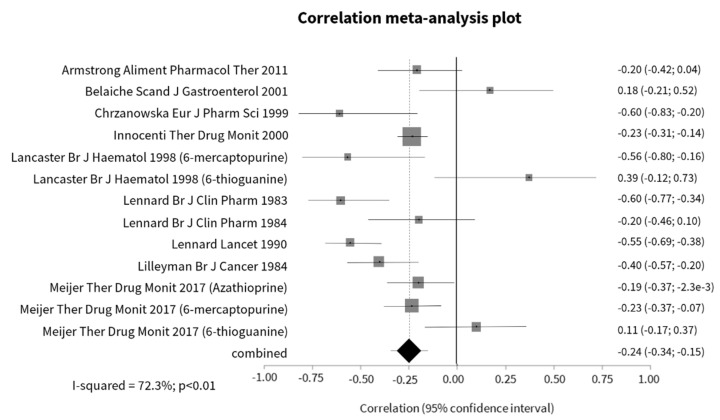
Correlation meta–analysis plot of 6-TGN levels and neutrophils.

A significant negative correlation between 6-TGN and neutrophils was registered in the pooled analysis of 10 studies [36,48,51,58,81,83,86,87,88,90] (Figure 4). This correlation was stronger in studies with conventional thiopurines (r = −0.27, 95% CI −0.36; −0.19; *p* < 0.01) than in those with 6-thioguanine, in which the correlation was in the inverse direction (r = 0.18, 95% CI 0.01; 0.34; *p* = 0.04). However, heterogeneity was substantial. Treatment duration was shown to contribute to a stronger correlation. In fact, the weighted correlation was greater in studies including patients with less and more than 8 weeks of treatment (−0.41 (95% CI −0.78; −0.04; *p* = 0.03) (*p* = 0.03; I^2^ = 78%) versus −0.26 (95% CI −0.37; −0.15; *p* < 0.01) (*p* < 0.01; I^2^ = 72%)). When the analysis was restricted to the three studies reporting using thiopurines in monotherapy, the correlation was not significant (r = −0.12, 95% CI −0.29; 0.05; *p* = 0.15) (*p* = 0.22; I^2^ = 33%), but all these studies included patients with more than 3 months of treatment.

##### Lymphopenia

In the pooled analysis of studies evaluating 6-TGN levels [51,54,78,94,100] and those evaluating 6-MMPR levels [94,100], no significant correlation was found between metabolites and lymphocytes (r = −0.02 and r = −0.18, respectively; *p* > 0.05 for both). However, when we limited the analysis to studies reporting the use of thiopurines in monotherapy, there was a weak but significant correlation between 6-TGN levels and lymphocytes (r = −0.15; 95% CI −0.26; −0.04; *p* < 0.01) (*p* = 0.73; I^2^ = 0%). The two studies evaluating correlation with 6-MMPR were also with patients on thiopurines monotherapy.

##### Thrombocytopenia

6-TGN

In the pooled analysis of five studies [34,36,48,81,108], no significant correlation was found between 6-TGN and platelets (Figure S9). However, when patients treated with 6-thioguanine were excluded, a weak but significant correlation between these variables was observed, with less heterogeneity between studies (r = 0.10, 95% CI −0.18; −0.02; *p* = 0.02) (*p* = 0.01; I^2^ = 64%). Similar results were obtained when the analysis was restricted to those studies reporting the use of thiopurines in monotherapy (r = −0.14; 95% CI −0.21; −0.06; *p* < 0.01) (*p* = 0.40; I^2^ = 0%).

#### 3.3.3. Liver Toxicity

##### Altered Liver Enzymes

6-TGN

In the two included studies evaluating this relationship, levels of 6-TGN were significantly lower in patients with liver toxicity (mean difference of −40.6 × 10^8^ RBC, 95% CI −69.99; −11.22; *p* < 0.01) (*p* = 0.67; I^2^ = 0%) [53,104]. We could also notice a significant negative correlation between 6-TGN levels and alanine aminotransferase (ALT), with a weighted mean correlation of −0.24 (95% CI −0.37; −0.11; *p* < 0.01) (*p* = 0.52; I^2^ = 0%) [76,95,108]. Of these studies, only one used thiopurines in monotherapy [104].

6-MMPR

**Figure 5:**
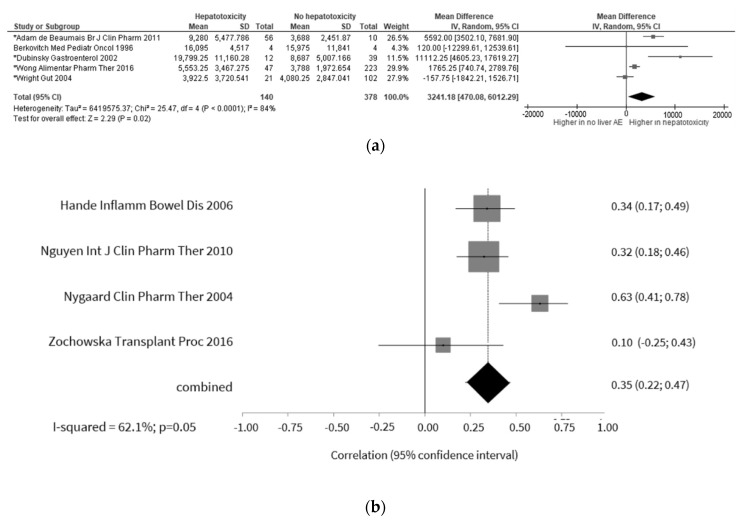

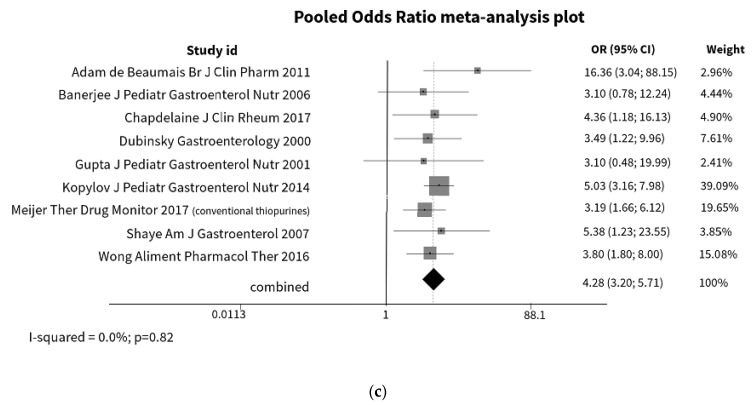
Relationship between 6-MMPR levels and hepatotoxicity. (**a**) Means difference meta-analysis plot (* studies in which mean was calculated from medians); (**b**) correlation meta-analysis plot; (**c**) odds ratio meta-analysis plot.

Our analysis evidenced that 6-MMPR concentrations were higher in patients with hepatotoxicity, with a mean difference of 3241.2 pmol/8 × 10^8^ RBC (Figure 5a) [44,53,68,104,106]. Nonetheless, the degree of heterogeneity between these studies was considerable. Restricting the analysis to studies reporting the use of thiopurines in monotherapy lead to non-statistically significant differences, also with considerable heterogeneity (mean difference of 5021.01 pmol × 10^8^ RBC, 95% CI −5987.12; 16,029.15; *p* = 0.37) (*p* < 0.01; I^2^ = 91%).

Regarding the relationship of 6-MMPR with ALT, we could observe a significant positive correlation, but with substantial heterogeneity, in four studies (Figure 5b) [77,93,95,108]. The results were similar when the analysis was restricted to studies with thiopurines monotherapy (r = 0.33; 95% CI 0.32; 0.34; *p* < 0.01) (*p* = 0.88; I^2^ = 0%).

Nine studies provided data for the calculation of a pooled OR [14,36,44,50,57,75,82,102,104]. It was possible to conclude that patients with 6-MMPR levels above the defined thresholds, ranging from 3615 to 5700 pmol/8 × 10^8^ RBC, were about four times more likely to develop hepatotoxicity (Figure 5c). When the analysis was restricted to studies reporting the use of thiopurines in monotherapy, the results were similar (OR 4.78; 95% CI 3.18; 7.19; *p* < 0.01) (*p* = 0.42; I^2^ = 0%).

6-MMPR/6-TGN Ratio

Our analysis showed that there was no significant association between 6-MMPR/6-TGN ratio (thresholds of 20 [57] and 24 [82]) and liver toxicity (OR 2.9, 95% CI 0.74; 11.55; *p* = 0.13) (*p* = 0.03; I^2^ = 80%).

##### Veno-occlusive Disease

In the pooled analysis of the two studies that evaluated the relationship between 6-TGN levels and the occurrence of veno-occlusive disease (VOD) [42,89], no difference was found in the 6-TGN levels in patients with this type of toxicity (mean difference 7.95 pmol/8 × 10^8^ RBC, 95% CI −118.57; 134.47; *p* = 0.90) (*p* = 0.29; I^2^ = 10%).

#### 3.3.4. Gastrointestinal Intolerance

##### 6-TGN

The pooled analysis of two studies revealed that patients with gastrointestinal intolerance presented higher levels of 6-TGN, with a mean difference of 201.46 pmol/8 × 10^8^ RBC (95% CI 16.86; 386.06; *p* = 0.03) (*p* = 0.23; I^2^ = 29%) [67,79].

#### 3.3.5. Pancreatitis, Infections and NRH

For each of these adverse events, only one study provided data on metabolites [66,80,109]. As such, it was not possible to perform meta-analysis, and these studies were not included.

## 4. Discussion

The wide use of thiopurines has been hindered by their inherent toxicity, which may also result in underdosing and lack of efficacy [5]. The measurement of thiopurines’ metabolites can give physicians a safer context for prescription, if the levels are kept in the therapeutic range and below toxic thresholds. However, the therapeutic range may differ depending on the disorder and on the indication. In IBD, the most cited optimal range is of ~230 to 400 pmol/8 × 10^8^ RBC, when thiopurines are used in monotherapy [12,13]. Still, thiopurines are also important in combination with anti–TNF drugs, and may be the most important factor in reducing the immunogenicity of these drugs [110,111]. In combination therapy with biologics, lower levels (105 to 125 pmol/8 × 10^8^ RBC) may be enough to improve anti-TNF pharmacokinetics [112,113].

In this study, we identified a relationship between thiopurines’ metabolites and several adverse events: (i) 6-TGN were associated with leukopenia, neutropenia and gastrointestinal intolerance, and inversely associated with liver toxicity; and (ii) 6-MMPR were associated with liver toxicity and early leukopenia. As myelosuppression has long been linked to 6-TGN, we calculated an optimal 6-TGN threshold (135 pmol/8 × 10^8^) for the occurrence of leukopenia. Although this cut-off is below the therapeutic levels for monotherapy with thiopurines in IBD, it is above the optimal cut-off for the levels demanded in combination therapy. This constitutes an additional argument for using lower doses of thiopurines when the drug is combined with infliximab. Regarding neutrophils and platelets, correlation with 6-TGN was only significant when the analysis was restricted to conventional thiopurines. Scientific evidence indicates that 6-TGN levels derived from 6-thioguanine have a different impact than those resulting from conventional thiopurines. Indeed, low doses of 6-thioguanine can lead to high 6-TGN levels without evidence of myelosuppression [114]. Some explanations can be pointed out for this fact. As most methods do not measure 6-TGN directly, but reduce it to thioguanine, the ingested 6-thioguanine is indistinguishable from 6-TGN, resulting in false high levels of 6-TGN if the drug is ingested close to the assay [115]. In addition, 6-MMPR are not produced with 6-thioguanine. Our results evidenced a possible association of early 6-MMPR assessment (i.e., in the first 8 weeks of treatment) with leukopenia. In previous studies, these metabolites were shown to be cytotoxic and to inhibit purine de novo synthesis, contributing to the antiproliferative properties of these drugs, responsible for both therapeutic and myelotoxic effects [116].

We have also confirmed a positive association of hepatoxicity with 6-MMPR, and a negative association with 6-TGN. In patients who metabolize thiopurines preferentially through the methylation pathway, generating high levels of 6-MMPR [13] (known as “shunters”), dose escalation will not always improve clinical outcomes. This explains thiopurines’ inefficacy despite optimal weight-based dosage [68]. Some strategies can be used to improve the metabolite profile in these patients: (i) dose-splitting regimen [117]; (ii) addition of allopurinol [1,117]; or (iii) use of 6-thioguanine instead of a conventional thiopurine [114].

Gastrointestinal intolerance to thiopurines is one of the most frequent adverse events with thiopurines treatment, causing many patients to abandon treatment [118]. Some authors postulated that this adverse event could be related to the nitro–imidazole compound released in AZA metabolism to form 6-MP [119]. In this way, 6-MP could be an adequate alternative to AZA treatment in patients experiencing gastrointestinal intolerance, as was demonstrated in some studies [119,120]. However, we have found that 6-TGN levels were associated with the occurrence of gastrointestinal intolerance. Accordingly, in one of the studies included in the analysis, switch of AZA to 6-MP was only tolerated in a small proportion of patients [79].

The reported higher sensitivity of Asian populations to thiopurines, when compared to Western populations, deserves particular consideration [121]. In this context and whenever possible, we performed subgroup-analysis according to the geographical origin of the studies. Generally, we could not evidence a significant influence of ethnicity on toxicity. Differences in metabolism are probably responsible for these “different sensitivities”. These results are strong arguments in favor of metabolites’ monitoring rather than the traditional weight-based strategy. In fact, even though metabolism may vary according to ethnicity, the significance of concentrations of metabolites is likely similar across populations.

The results of this meta-analysis were impacted by several limitations. Most studies were retrospective, with small samples and, in many of them, the evaluation of the relationship between metabolites and toxicity was a secondary outcome. As such, toxicity events were possibly not always registered, and samples were often underpowered for conclusive results. The mean global quality score was only 58.9%, reflecting these factors. In most cases, studies displayed a high degree of heterogeneity and the definitions of toxic events were inconsistent, suggesting that the results should be interpreted with caution. This heterogeneity was, in part, caused by the different methods applied for the measurement of metabolites. Even though conversion factors for 6-TGN have been described to uniformize values, small variations in protocols can lead to significant differences in 6-TGN concentrations [1,31,35]. Levels of 6-MMPR are reported as being similar in different assays [35,36,37], but this causes a problem in the definition of optimal values for 6-MMPR/6-TGN ratios. Cut-offs obtained by a specific assay are not interchangeable [31] and thus the calculated optimal threshold for leukopenia should be used with caution in clinical practice. In the future, the standardization of procedures for the assessment of metabolites is of upmost importance [41,117]. Another limitation of this analysis is that most studies included patients with more than 8 weeks of treatment, but most adverse events with thiopurines occur in the first weeks/months [4,5,79]. Hence, a large proportion of patients had their doses of drug reduced (or even discontinued) at the time of the study. The strength of association between metabolites and adverse events could be stronger if more studies included patients in the beginning of thiopurine treatment. In fact, Wong et al. demonstrated that the assessment of metabolites at week 1 could predict the later occurrence of leukopenia and hepatotoxicity [104,105]. Some authors reduce the doses of thiopurines or change the treatment strategy in patients with high 6-MMPR based on similar experiences in their clinical practice, but this strategy is yet to be validated. [68] To perform this meta-analysis, we converted median values (reported in most studies) to mean values. This procedure could have introduced some error in the final analysis. However, whenever possible, we performed sub-analysis by “provided vs. calculated means”, with no impact on the results. To finalize, many studies were excluded from the meta-analysis for not providing enough data for calculations. Most of these studies mentioned the absence of significant relationships between metabolites and adverse events, which might result in a positive results bias.

As many thiopurine-associated adverse events are related to the level of metabolites, physicians should take this information into account for dose selection, to achieve the best compromise between efficacy and toxicity. The importance of establishing a clear relationship between metabolite levels and toxicity may also be of value in patients receiving concomitant medications with similar toxicity profiles. In these cases, metabolites’ measurement will help to determine the culprit. The same applies to disorders in which the clinical presentation resembles drug toxicity, as in the case of AIH flares. If the context enables TPMT and nudix hydrolase-15 (NUDT15) screening before starting the treatment to inform on eligibility or drug dosage, subsequent adjustments can be guided by the measurement of metabolites, in a tiered approach [117]. However, at this point, these strategies should be used as adjuncts in clinical practice and cannot yet replace blood and clinical monitoring for early detection of toxicity. A more personalized medicine should overcome the traditional weight-based dosing of thiopurines and rely more on TDM. Still, higher quality studies are needed to confirm this strategy.

## Figures and Tables

**Table 1:** Description of the 72 studies included in the meta-analysis.

Study	Study Design	Study Population	Population with Evaluation of Metabolites and Toxicity (If Different from Total Number)	Disease	Treatment Regimen and Duration	Concomitant Medication	Measured Metabolites	Measurements Per Patient (If Multiple, Used Value)	Method	QAT Score (%)
Adam de Beaumais et al., Br J Clin Pharm 2011 [44]	Prospective	66 patients, pediatric	Same	Leukemia	6-MP >4 weeks	Methotrexate	6-MMPR	Multiple (average of all samples per patient)	Dervieux and Boulieu	67.8
Almer et al., Dig Liver Dis 2009 [45]	Prospective	23 patients, adult	Same	IBD	6-TG From the start (timeline not discriminated)	Mesalazine, steroids and antibiotics.	6-TGN	Multiple (maximum value of metabolites)	Lennard and Singleton	40.5
Alvarez Beltran et al., An Pediatr 2009 [46]	Retrospective	107 patients, pediatric	18 patients	IBD and AIH	AZA >2 months	NS	6-TGN and 6-MMPR	NS	NS	42.9
Andoh et al., J Gastroenterol Hepatol 2008 [47]	Retrospective	83 patients, adult	Same	IBD	AZA + 6-MP >4 months	Mesalazine	6-TGN	NS	Erdmann	41.7
Armstrong et al., Aliment Pharmacol Ther 2011 [48]	Retrospective	70 patients, pediatric	Same	IBD	AZA + 6-MP >3 months	Mesalazine	6-TGN	Multiple (NS)	Dervieux and Boulieu	38.1
Ban et al., J Gastroenterol 2010 [49]	Prospective	279 patients, pediatric and adult	130 patients	IBD	AZA + 6-MP NS	Mesalazine	6-TGN	NS	Erdmann	54.7
Banerjee et al., J Pediatr Gastroenterol Nutr 2006 [50]	Retrospective	101 patients, pediatric	64 patients	IBD	AZA + 6-MP >6 months	Mesalazine, steroids, antibiotics and infliximab	6-MMP	Multiple (evaluation per-sample)	Prometheus	65.5
Belaiche et al., Scand J Gastroenterol 2001 [51]	Prospective	28 patients, adult	Same	IBD	AZA + 6-MP >3 months	Steroids	6-TGN	Single	Lennard and Singleton	52.4
Bergan et al., Transplantation 1994 [52]	Prospective	65 patients, pediatric and adult	62 patients	Renal transplant	AZA Initial 40 days	Ciclosporin and steroids	6-TGN	Multiple (division in 2 groups: patients with all 6-TGN below threshold, and patients with at least one 6-TGN measure above threshold)	Lennard	67.8
Berkovitch et al., Med Pediatr Oncol 1996 [53]	Retrospective	29 patients, pediatric	8 patients	Leukemia	6-MP NS	Chemotherapy	6-MMPR	Single	Lennard and Singleton	40.5
Boulieu et al., Br J Clin Pharm 1997 [54]	Prospective	47 patients, adult	Same	Transplant	AZA >3 months	Cyclosporine and steroids	6-TGN	Single	Dervieux and Boulieu	39.3
Boulieu et al., Adv Exp Med Biol 2000 [55]	Prospective	27 patients, adult	Same	Transplant	AZA >3 months	Steroids and Cyclosporine	6-TGN and 6-MMPR	Single	Dervieux and Boulieu	45.2
Broekman et al., Aliment Pharm Ther 2017 [56]	Prospective	695 patients, adult	301 patients	IBD	AZA + 6-MP Week 8	Mesalazine, steroids, biologics	6-TGN and 6-MMPR	Single	Lennard and Singleton	76.2
Chapdelaine et al., J Clin Rheumatol 2017 [57]	Retrospective	71 patients, adult	Same	Rheumatologic disorders	AZA NS	NS	6-TGN and 6-MMPR	Multiple (NS)	Lennard and Singleton	65.5
Chrzanowska et al., Eur J Pharm Sci 1999 [58]	Prospective	19 patients, pediatric	Same	Leukemia	6-MP >1 month	Methotrexate	6-TGN and 6-MMPR	Single	Lennard and Singleton	63.1
Chrzanowska et al., Ther Drug Monit 1999 [59]	Prospective	37 patients, pediatric and adult	Same	Transplant and glomerulonephritis	AZA >1 month	Cyclosporine and steroids	6-TGN	Single	Lennard and Singleton	46.4
Cuffari et al., Gut 1996 [60]	Prospective	25 patients, pediatric	Same	IBD	6-MP >4 months	Low-dose steroids	6-TGN and 6-MMP	Single	Lennard and Singleton	61.9
Cuffari et al., Dig Dis Sci 2004 [61]	Prospective	46 patients, adult	Same	IBD	AZA NS	Mesalazine and “other medications” allowed (NS)	6-TGN	NS	Lennard and Singleton	60.7
Dassopoulos et al., Aliment Pharmacol Ther 2014 [62]	Prospective	50 patients, pediatric and adult	Same	IBD	AZA Week 4 to week 44	Steroids; other immunosuppressants not allowed	6-TGN	NS	Prometheus	71.4
De Boer et al., World J Gastroenterol 2005 [63]	Retrospective	95 patients, adult	55 patients	IBD	TG >4 weeks	Other immunosuppressants not allowed (cyclosporine, infliximab, methotrexate, thalidomide)	6-TGN	Single	Lennard and Singleton	41.7
Derijks et al., Eur J Gastroen Hepat 2003 [64]	Prospective	32 patients, adult	Same	IBD	6-TG Week 1 to week 8	Other immunosuppressants not allowed	6-TGN	Multiple (correlation per event)	Lennard and Singleton	51.2
Derijks et al., Ther Drug Monit 2004 [65]	Prospective	30 patients, adult	17 patients	IBD	6-MP Week 1 to week 8	Mesalazine; other immunosuppressants not allowed	6-TGN	Multiple (level of metabolites at the time of AE; for the non-AE group, levels at week 8)	Lennard and Singleton	64.3
Dervieux et al., Leukemia 2001 [66]	Prospective	78 patients, pediatric	25 patients	Leukemia	6-MP At least >4 weeks	Methotrexate	6-TGN	Multiple (steady-state concentration)	Dervieux and Boulieu	51.2
Ding et al., Inflamm Bowel Dis 2012 [67]	Prospective	120 patients, pediatric and adult	104 patients	IBD	AZA + 6-MP Week 8	Mesalazine and infliximab; methotrexate and cyclosporine not allowed	6-TGN	Single	Dervieux and Boulieu	76.2
Dubinsky et al., Gastroenterology 2000 [14]	Prospective	92 patients, pediatric	Same	IBD	AZA + 6-MP >4 months	Mesalazine	6-MMPR	Multiple (NS)	Lennard and Singleton	66.7
Dubinsky et al., Gastroenterology 2002 [68]	Retrospective	51 patients, pediatric and adult	Same	IBD	AZA + 6-MP >3 months	Mesalazine and steroids	6-MMPR	Multiple (median values)	Prometheus	70.2
Dubinsky et al., Gastroenterology 2003 [69]	Retrospective	111 patients, pediatric and adult	Same	IBD	6-TG 1 to 28 months	Steroids, mesalazine and infliximab	6-TGN	NS	Prometheus	54.8
Fangbin et al., Medicine 2016 [40]	Prospective	132 patients, adult	Same	IBD	AZA Week 1 to week 48	Mesalazine and infliximab	6-TGN	Multiple (maximum tgn at the time of AE) *For optimal cut-off calculus, all 471 samples were used*	Dervieux and Boulieu for 6-TGN and Lennard and Singleton for 6-MMP	64.3
Fei et al., Front Pharmacol 2018 [70]	Retrospective	87 patients, adult	Same	Multiple (NS)	AZA >2 months	Medications interfering with metabolite levels and/or causing leukopenia were excluded (cycloscporine, tacrolimus, mesalazine, allopurinol, diuretics)	6-TGN	Single	Dervieux and Boulieu	70.3
Feng et al., J Gastroenterol Hepatol 2018 [71]	Retrospective	252 patients, adult	Same	IBD	AZA >3 months	Mesalazine and antibiotics; biologics, thalidomide and steroids not allowed	6-TGN	Multiple (evaluation per-sample)	Dervieux and Boulieu	73.8
Ferucci et al., Can J Gastroenterol 2011 [72]	Retrospective	71 patients, adult	48 patients	AIH	AZA NS	NS	6-TGN and 6-MMPR	Multiple (most recent value available)	Prometheus	70.2
Ganping et al., Int J Pharmacol 2008 [73]	Prospective	10 patients, pediatric	Same	Leukemia	6-MP >2 months	Methotrexate	6-TGN	Multiple (level of metabolites measured 7 days before laboratorial evaluation of AE)	Lennard and Singleton	39.3
Gardiner et al., Clin Gastroenterol Hepatol 2008 [74]	Prospective	69 patients, >16 years old	61 patients	IBD	AZA + 6-MP Month 1 to month 9	No patient was excluded based on concomitant medication; concomitant drugs NS	6-TGN and 6-MMPR	Multiple (level of metabolites within 2 days of stopping treatment in the AE group; for the non-AE group, values at month 1)	Dervieux and Boulieu	66.7
Gupta et al., J Pediatr Gastroenterol Nutr 2001 [75]	Retrospective	101 patients, pediatric	Same	IBD	AZA + 6-MP >4 months	NS	6-TGN and 6-MMPR	Multiple (NS)	Prometheus	52.4
Halonen et al., Pediatr Blood Cancer 2006 [76]	Prospective	16 patients, pediatric	Same	Leukemia	6-MP NS	Chemotherapy	6-TGN	Multiple (average of all samples per patient)	Bruunshuus	59.5
Hande et al., Inflamm Bowel Dis 2006 [77]	Retrospective	126 patients, pediatric and adult	121 patients	IBD	AZA + 6-MP >3 months	Mesalazine; steroids, infliximab and other immunosuppressants not allowed	6-TGN and 6-MMPR	Multiple (most recent values)	Prometheus	73.8
Heerasing et al., Intern Med J 2016 [78]	Retrospective	67 patients, NS	Same	IBD	AZA + 6-MP NS	NS	6-TGN	NS	NS	42.9
Hindorf et al., Aliment Pharmacol Ther 2006 [79]	Retrospective	364 patients, pediatric and adult	266 patients	IBD	AZA + 6-MP + 6-TG NS	Only mesalazine and steroids	6-TGN and 6-MMPR	Multiple (at the time of AE; for the non-AE group, last result available)	Lennard and Singleton	81.0
Hindorf et al., Gut et al. 2006 [80]	Prospective	60 patients, adult	54 patients	IBD	AZA + 6-MP Week 1 to week 20	Mesalazine, steroids, infliximab	6-TGN and 6-MMPR	Multiple (maximum value of metabolites)	Lennard and Singleton	59.5
Innocenti et al., Ther Drug Monit 2000 [81]	Prospective	19 patients, pediatric	Same	Leukemia	6-MP >3 months	Chemotherapy	6-TGN	Multiple (evaluation per-sample)	Lennard and Singleton	65.5
Kopylov et al., J Pediatr Gastroenterol Nutr 2014 [82]	Prospective	237 patients, pediatric	Same	IBD	AZA + 6-MP >3 months	Mesalazine and steroids; methotrexate and biologics not allowed	6-MMPR	Multiple (evaluation per-sample)	Lennard and Singleton	63.1
Lancaster et al., Br J Haematol 1998 [83]	Prospective	46 patients, pediatric	37 patients	Leukemia	6-MP + 6-TG Measurements available from at least week 3 (not mentioned if for all patients)	Chemotherapy	6-TGN	Multiple (earliest essay)	Lennard and Singleton	53.6
Lee at al., Inflamm Bowel Dis 2015 [84]	Retrospective	137 patients, pediatric	Same	IBD	AZA >2 months	Mesalazine, steroids, infliximab	6-TGN	Multiple (evaluation per-sample)	Dervieux and Boulieu	63.1
Lee et al., PLoS One 2017 [85]	Retrospective	165 patients, adult	Same	IBD	AZA + 6-MP >3 months	Steroids and mesalazine; patients using anti-TNF were excluded	6-TGN and 6-MMPR	NS	Dervieux and Boulieu	67.8
Lennard et al., Br J Clin Pharm 1983 [86]	Prospective	22 patients, pediatric	Same	Leukemia	6-MP >4 weeks	Chemotherapy	6-TGN	Multiple (level of metabolites measured 14 days before laboratorial evaluation)	Lennard and Singleton	70.2
Lennard et al., Br J Clin Pharm 1984 [87]	Prospective	54 patients, NS	46 patients	Transplant	AZA >6 months	Steroids	6-TGN	Multiple (evaluation per-sample)	Lennard and Singleton	51.2
Lennard et al., Lancet 1990 [88]	Retrospective	225 patients, pediatric	82 patients	Leukemia	6-MP >2 months	Chemotherapy	6-TGN	Single	Lennard and Singleton	46.4
Lennard et al., Clin Pharm Ther 2006 [89]	Prospective	1492 patients, pediatric	134 patients	Leukemia	TG >7 days	Chemotherapy	6-TGN	Single	Lennard and Singleton	67.8
Lilleyman et al., Br J Cancer 1984 [90]	Prospective	22 patients, pediatric	Same	Leukemia	6-MP >7 months	Chemotherapy	6-TGN	Multiple (level of metabolites measured 14 days before laboratorial evaluation)	Lennard and Singleton	63.1
Liu et al., Scand J Gastroenterol 2016 [91]	Prospective	69 patients, adult	Same	IBD	AZA >3 months	Steroids and Infliximab	6-TGN	NS	Dervieux and Boulieu	69.1
Meijer et al., J Gastroenterol Hepatol 2017 [37]	Retrospective	24 patients, adult	Same	IBD	AZA + 6-MP Median 11 weeks (IQR 6-46)	Steroids; no mention to additional medication	6-MMPR	Multiple (level of metabolites within 3 days of AE)	Lennard and Singleton	50.0
Meijer et al., Ther Drug Monit 2017 [36]	Retrospective	424 patients, adult	Same	IBD, AIH and celiac disease	AZA + 6-MP + TG NS	NS	6-TGN and 6-MMP	Multiple (evaluation per-sample when laboratory data within 3 days are available)	Dervieux and Boulieu (but converted to Lennard by a factor of 2.6)	63.1
Melaouhia et al., Therapie 2013 [92]	Prospective	50 patients, adult	Same	IBD	AZA >12 months	Mesalazine and steroids	6-TGN and 6-MMPR	Multiple (NS)	Dervieux and Boulieu	44.1
Nguyen et al., Int J Clin Pharm 2010 [93]	Retrospective	71 patients, pediatric	Same	IBD	AZA >1 year	Mesalazine	6-TGN and 6-MMPR	Multiple (evaluation per-sample)	Dervieux and Boulieu	38.1
Nguyen et al., Ther Drug Monitor 2010 [94]	Retrospective	28 patients, pediatric	Same	AIH	AZA >3 months	Steroids	6-TGN and 6-MMPR	Multiple (NS)	Dervieux and Boulieu	48.8
Nygaard et al., Clin Pharm Ther 2004 [95]	Retrospective	43 patients, pediatric	Same	Leukemia	6-MP >4 weeks	Methotrexate	6-TGN and 6-MMPR	Multiple (average of all samples per patient)	Erdmann	54.7
Odahara et al., PLoS One 2015 [96]	Prospective	48 patients, adult	Same	IBD	AZA NS	Mesalazine and Infliximab	6-TGN	Multiple (level of metabolites at the time of AE; for the non-AE group, mean-value between weeks 8 and 52)	Lennard and Singleton	59.5
Ohtsuka et al., J Gastroenterol Hepatol 2010 [97]	Retrospective	51 patients, pediatric	Same	IBD	AZA + 6-MP >3 weeks	Mesalazine and steroids	6-TGN	Multiple (evaluation per-sample)	Erdmann	40.5
Ooi et al., Aliment Pharm Ther 2007 [98]	Retrospective	56 patients, pediatric	Same	IBD	AZA + 6-MP >1 month	Steroids > 10 mg/day, infliximab, tacrolimus, methotrexate and cyclosporine not allowed	6-TGN	Multiple (evaluation per-sample)	Lennard and Singleton	53.6
Pranzatelli et al., J Clin Exp Immunol 2017 [99]	Retrospective	10 patients, pediatric	Same	Opsoclonus-myoclonus	6-MP >7 months	Adrenocorticotrophic hormone, intravenous immunoglobulin and steroids	6-TGN	Multiple (NS)	Prometheus	53.6
Rae et al., J Neuroimmunol 2016 [100]	Prospective	19 patients, adult	Same	Myasthenia gravis	AZA ≥52 weeks	Steroids	6-TGN and 6-MMP	NS	Dervieux and Boulieu	57.1
Sandborn et al., Gastroenterology 1999 [43]	Prospective	96 patients, adult	Same	IBD	AZA From week 0.2 to week 16	Steroids	6-TGN	Multiple (evaluation per sample)	Erdmann	88.1
Schmiegelow et al., Cancer Chemother Pharmacol 1990 [101]	Prospective	31 patients, pediatric	Same	Leukemia	6-MP >5 weeks	Chemotherapy	6-TGN	Multiple (mean of measurements)	Bruunshuus	52.4
Shaye et al., Am J Gastroenterol 2007 [102]	Retrospective	173 patients, adult	Same	IBD	AZA + 6-MP >1 month	Mesalazine	6-MMPR	NS	Prometheus	59.5
Stoneham et al., Br J Haematol 2003 [42]	Retrospective	99 patients, pediatric	Same	Leukemia	6-MP + TG Week 4	NS	6-TGN	Single	Lennard and Singleton	34.5
Thomas et al., Inflamm Bowel Dis 2003 [34]	Prospective	166 patients, adult	158 patients	IBD	AZA + 6-MP >3 months	Sulfassalazine	6-TGN	Single	Erdmann	63.1
Wojtuskiewicz et al., Nucleos Nucleot Nucl 2014 [103]	Prospective	236 patients, pediatric and adult	41 patients	Leukemia	6-MP Measurements from week 25 to 109	Chemotherapy	6-TGN	Multiple (metabolite levels at week 25)	Keuzenkamp	63.1
Wong et al., Aliment Pharmacol Ther 2016 [104]	Prospective	270 patients, adult	Same	IBD	AZA + 6-MP Week 1	Mesalazine, steroids and anti-TNF	6-MMPR	Single	Lennard and Singleton	82.1
Wong et al., J Crohn Colitis 2017 [105]	Prospective	194 patients, adult	Data for 194 patients available; data from 181 patients were used in the means comparison and pooled OR analyses (exclusion of patients using anti-TNF)	IBD	AZA + 6-MP Week 1	Mesalazine and steroids; (patients using anti-TNF were excluded from means comparison and pooled OR analysis; for calculation of an optimal cutoff, data from all patients were used)	6-TGN and 6-MMPR	Single	Lennard and Singleton	82.1
Wright et al., Gut 2004 [106]	Prospective	159 patients, NS	123 patients	IBD	AZA >4 months	Mesalazine and steroids	6-MMPR	Multiple (average of all samples per patient)	Lennard and Singleton	78.6
Yarur et al., J Clin Gastroenterol 2018 [107]	Retrospective	87 patients, adult	Same	IBD	AZA + 6-MP >8 weeks	Mesalazine; biologics, cyclosporine and tacrolimus not allowed	6-TGN	Multiple (nadir values, median and peak available; analysis made with median)	NS	63.1
Zochowska et al., Transplant Proc 2016 [108]	NS	33 patients, adult	Same	Transplant	AZA NS	Calcineurin inhibitors, steroids	6-TGN and 6-MMPR	NS	Other (description provided)	51.2

6-MP: 6-mercaptopurine; 6-MMPR: 6 methylmercaptopurine ribonucleotides; 6-TG: 6-thioguanine; 6-TGN: 6-thioguanine nucleotides; AE: adverse events; AIH: autoimmune hepatitis; AZA: azathioprine; IBD: inflammatory bowel disease; NS: non-specified; OR: Odds ratio; QAT: quality assessment tool.

## Supplementary Materials

The following are available online at https://www.mdpi.com/2077-0383/9/7/2216/s1, Figure S1: Relationship between 6-TGN levels and overall toxicity: mean difference forest plot (AE—adverse events) with sub-analysis by age group; Figure S2: Relationship between 6-MMPR levels and overall toxicity: mean difference forest plot (AE—adverse events); Figure S3: Relationship between 6-TGN levels and overall myelotoxicity: mean difference forest plot with sub-analysis by duration of treatment; Figure S4: Relationship between 6-TGN levels and hemoglobin: correlation meta-analysis plot and sub-analysis of studies including patients with inflammatory bowel disease; Figure S5: Mean difference forest plot of 6-MMPR levels and leukopenia: (**a**) sub-analysis by method (**b**) sub-analysis by treatment duration; Figure S6: Relationship between 6 MMPR levels and leukopenia: correlation meta-analysis plot; Figure S7: Relationship between 6-MMPR/6-TGN ratio and leukopenia: mean difference forest plot; Figure S8: Relationship between neutrophils levels and 6-TGN levels: mean difference forest plot; Figure S9: Correlation meta-analysis plot of 6-TGN and platelets; Table S1: Type and definition of toxicities evaluated in each study included in the meta-analysis; Table S2: Measures of performance of different 6-TGN cut-offs for leukopenia occurrence (calculated with the method described in Steinhauser S, Schumacher M, Rucker G. Modelling multiple thresholds in meta-analysis of diagnostic test accuracy studies. BMC medical research methodology. 2016;16(1):97).
