# Supplementary material for: Thiopurines’ Metabolites and Drug Toxicity: A Meta-Analysis
Source: J Clin Med. 2020 Jul 13;9(7):2216. doi: 10.3390/jcm9072216 (PMC7408995; doi:10.3390/jcm9072216)
Supplement: Supplementary file 1 [file jcm-09-02216-s001.zip › Table S1.docx]

**Supplementary Table 1: Type and definition of toxicities evaluated in each study included in the meta-analysis**

| **Study** | **Toxicity** | **Definition** | **Threshold values** | | |
| --- | --- | --- | --- | --- | --- |
|  |  |  | **6-TGN*** | **6-MMP**** | **Ratio** |
| Adam de Beaumais et al, Br J Clin Pharm 2011 | Hepatotoxicity | ALP, bilirubin, GGT, AST or ALT > ULN | NA | 4884 | NA |
| Almer et al, Dig Liver Dis 2009 | Toxicity in general | Adverse events/side effects that led to discontinuation of the drug or dose reduction | NA | NA | NA |
| Alvarez Beltran et al, An Pediatr 2009 | Toxicity in general | Myelotoxicity, hypertransaminemia (transaminases > 2 x ULN) or pancreatitis | NA | NA | NA |
|  | General myelotoxicity | Leukopenia | 450 | NA | NA |
|  | Leukopenia | WBC < 4 x 10^9^/L | NA | 3525 | NA |
| Andoh et al, J Gastroenterol Hepatol 2008 | Leukopenia | (correlation) | NA | NA | NA |
| Armstrong et al, Aliment Pharmacol Ther 2011 | General myelotoxicity | Leukopenia (as below) or neutropenia (as below) | NA | NA | NA |
|  | Anaemia | (correlation) | NA | NA | NA |
|  | Leukopenia | WBC < 4 x 10^9^/L | NA | NA | NA |
|  | Neutropenia | ANC <2 x 10^9^/L | NA | NA | NA |
|  | Thrombocytopenia | (correlation) | NA | NA | NA |
| Ban et al, J Gastroenterol 2010 | Leukopenia | (correlation) | NA | NA | NA |
| Banerjee et al, J Pediatr Gastroenterol Nutr 2006 | Hepatotoxicity | ALT or AST > 2 x ULN | NA | 5700 | NA |
| Belaiche et al, Scand J Gastroenterol 2001 | Leukopenia | (correlation) | NA | NA | NA |
|  | Lymphopenia | (correlation) | NA | NA | NA |
|  | Neutropenia | (correlation) | NA | NA | NA |
| Bergan et al, Transplantation 1994 | Neutropenia | (continuous variable) | 200 | NA | NA |
| Berkovitch et al, Med Pediatr Oncol 1996 | Hepatotoxicity | Jaundice, tender enlarged liver and elevated liver enzymes (AST, ALT and GGT) | NA | NA | NA |
| Boulieu et al Br J Clin Pharm 1997 | Anaemia | (correlation) | NA | NA | NA |
|  | Leukopenia | (correlation) | NA | NA | NA |
|  | Lymphopenia | (correlation) | NA | NA | NA |
| Boulieu et al, Adv Exp Med Biol 2000 | Leukopenia | (correlation) | NA | NA | NA |
| Broekman et al Aliment Pharm Ther 2017 | Leukopenia | WBC < 3 x 10^9^/L | NA | NA | NA |
| Chapdelaine et al, J Clin Rheumatol 2017 | Leukopenia | WBC < 4 x 10^9^/L | NA | NA | NA |
| Chrzanowska et al, Eur J Pharm Sci 1999 | General myelotoxicity | Leukopenia (as below) and/or neutropenia (as below) | 450 | 5700 | 20 |
|  | Leukopenia | WBC < 3.5 x 10^9^/L | 450 | 3525 | 20 |
|  | Neutropenia | ANC < 1.5 x 10^9^/L | NA | NA | NA |
| Chrzanowska et al, Ther Drug Monit 1999 | Leukopenia | (correlation) | NA | NA | NA |
| Cuffari et al, Gut 1996 | Toxicity in general | Myelotoxicity, pancreatitis and hepatitis | NA | NA | NA |
| Cuffari et al, Dig Dis Sci 2004 | Leukopenia | (correlation) | NA | NA | NA |
| Dassopoulos et al, Aliment Pharmacol Ther 2014 | Leukopenia | (correlation) | NA | NA | NA |
| De Boer et al, World J Gastroenterol 2005 | Toxicity in general | Any adverse event leading to discontinuation of thioguanine | NA | NA | NA |
| Derijks et al, Eur J Gastroenterol Hepatol 2003 | Leukopenia | (correlation) | NA | NA | NA |
| Derijks et al, Ther Drug Monit 2004 | Leukopenia | WBC < 4 x 10^9^/L | 450 | NA | NA |
| Dervieux et al, Leukemia 2001 | Leukopenia | (correlation) | NA | NA | NA |
| Ding et al, Inflamm Bowel Dis 2012 | Leukopenia | WBC < 3.5 x 10^9^/L | NA | NA | NA |
|  | Intolerance | Hypogeusia, nausea and vomiting | NA | NA | NA |
| Dubinsky et al, Gastroenterology 2000 | Hepatotoxicity | AST or ALT > 2 x ULN | NA | 5700 | NA |
| Dubinsky et al Gastroenterology 2002 | Hepatotoxicity | ALT or AST > 2 times ULN | NA | NA | NA |
| Dubinsky et al Gastroenterology 2003 | Toxicity in general | Abnormal liver chemistry values and/or evidence of hematologic toxicity | NA | NA | NA |
| Fangbin et al, Medicine 2016 | Leukopenia | WBC < 3.5 x 10^9^/L | *162* | NA | NA |
| Fei et al, Front Pharmacol 2018 | Leukopenia | WBC < 3.5 x 10^9^/L | NA | NA | NA |
| Feng et al, J Gastroenterol Hepatol 2018 | Toxicity in general | Gastric intolerance, flu-like symptoms, pancreatitis, hepatitis, rash, myelotoxicity, infection and nonspecific side effects requiring drug withdrawal or dose reduction | *137* | NA | NA |
| Ferucci et al, Can J Gastroenterol 2011 | Leukopenia | WBC < 4 x 10^9^/L | *368* | NA | NA |
| Ganping et al, Int J Pharmacol 2008 | Leukopenia | WBC < 3.5 x 10^9^/L | 450 | NA | NA |
| Gardiner et al, Clin Gastroenterol Hepatol 2008 | Toxicity in general | Any adverse event leading to drug discontinuation (including hepatotoxicity – transaminases > 2 x ULN -, pancreatitis – severe abdominal pain and amylase > 3 x ULN -, myelosuppression - WBC < 3 x 10^9^/L and/or ANC < 2 x 10^9^/L -, flu-like/hypersensitivity illness – arthralgia, myalgia, fever and/or rash -, or nausea/vomiting) | NA | NA | NA |
| Gupta et al, J Pediatr Gastroenterol Nutr 2001 | Leukopenia | WBC < 4 x 10^9^/L | NA | NA | NA |
| Halonen et al, Pediatr Blood Cancer 2006 | Hepatotoxicity | (correlation) | NA | NA | NA |
| Hande et al, Inflamm Bowel Dis 2006 | Leukopenia | (correlation) | NA | NA | NA |
|  | Hepatotoxicity | (correlation) | NA | NA | NA |
| Heerasing et al, Intern Med J 2016 | Lymphopenia | (correlation) | NA | NA | NA |
| Hindorf et al, Aliment Pharmacol Ther 2006 | Toxicity in general | Myelotoxicity (as below); hepatotoxicity (AST or ALT > 5 x ULN or ALP > 3 x ULN); pancreatitis (severe abdominal pain + rise in serum amylase levels); allergic/systemic reactions (arthralgia, myalgia, fever, rash, general malaise and other non-specific reactions); gastrointestinal intolerance (as below) | 400 | NA | NA |
|  | General myelotoxicity | Anaemia (Hb < 120 g/L); leukopenia (<3 x 10^9^/L); neutropenia (ANC <1.5 x 10^9^/L); thrombocytopenia (platelet count < 100 x 10^9^/L) | NA | NA | NA |
|  | Intolerance | Abdominal pain, nausea, vomiting and diarrhoea | NA | NA | NA |
| Hindorf et al, Gut et al 2006 | Toxicity in general | Any adverse event leading to drug discontinuation or dose reduction | NA | NA | NA |
|  | General myelotoxicity | Leukopenia (WBC < 3 x 10^9^/L), neutropenia (ANC< 1.5 x 10^9^/L), thrombocytopenia (platelet count < 100 x 10^9^/L) | NA | 11450 | NA |
| Innocenti et al, Ther Drug Monit 2000 | Anaemia | (correlation) | NA | NA | NA |
|  | Leukopenia | (correlation) | NA | NA | NA |
|  | Neutropenia | (correlation) | NA | NA | NA |
|  | Thrombocytopenia | (correlation) | NA | NA | NA |
| Kopylov et al, J Pediatr Gastroenterol Nutr 2014 | Hepatotoxicity | Elevation of liver enzymes > 2 x ULN | NA | 5700 | 24 |
| Lancaster et al, Br J Haematol 1998 | Neutropenia | (correlation) | NA | NA | NA |
| Lee at al, Inflamm Bowel Dis 2015 | Leukopenia | WBC < 3 x 10^9^/L | NA | NA | NA |
|  | Neutropenia | ANC < 1.5 x 10^9^/L | NA | NA | NA |
| Lee et al, PLoS One 2017 | Leukopenia | WBC < 3 x 10^9^/L | *127* | NA | NA |
| Lennard et al, Br J Clin Pharm 1983 | Neutropenia | (correlation) | NA | NA | NA |
| Lennard et al, Br J Clin Pharm 1984 | Leukopenia | (correlation) | NA | NA | NA |
|  | Neutropenia | (correlation) | NA | NA | NA |
| Lennard et al, Lancet 1990 | Neutropenia | (correlation) | NA | NA | NA |
| Lennard et al, Clin Pharm Ther 2006 | Veno-occlusive disorder | At least 2 of the following: tender hepatomegaly, ascites, hyperbilirubinemia (> 17 µmol/L) or thrombocytopenia (platelet count < 75 x 10^9^/L) in the absence of neutropenia, of at least 4 weeks’ duration. | NA | NA | NA |
| Lilleyman et al, Br J Cancer 1984 | Neutropenia | (correlation) | NA | NA | NA |
| Liu et al, Scand J Gastroenterol 2016 | Leukopenia | WBC < 3.5 x 10^9^/L | NA | NA | NA |
| Meijer er al, J Gastroenterol Hepatol 2017 | Leukopenia | (correlation) | NA | NA | NA |
| Meijer et al, Ther Drug Monit 2017 | General myelotoxicity | Leukopenia (WBC < 3 x 10^9^/L) or thrombocytopenia (platelet count < 75 x 10^9^/L) | 450 | 5700 | NA |
|  | Anaemia | (correlation) | NA | NA | NA |
|  | Leukopenia | (correlation) | 450 | NA | NA |
|  | Neutropenia | (correlation) | NA | NA | NA |
|  | Thrombocytopenia | (correlation) | NA | NA | NA |
|  | Hepatotoxicity | AST, ALT, ALP or GGT > 2 x ULN | NA | 5700 | NA |
| Melaouhia et al, Therapie 2013 | Leukopenia | WBC < 3.5 x 10^9^/L | NA | NA | NA |
| Nguyen et al, Int J Clin Pharm 2010 | Anaemia | (correlation) | NA | NA | NA |
|  | Hepatotoxicity | (correlation) | NA | NA | NA |
| Nguyen et al, Ther Drug Monitor 2010 | Anaemia | (correlation) | NA | NA | NA |
|  | Leukopenia | WBC < 4 x 10^9^/L | NA | NA | NA |
|  | Lymphopenia | (correlation) | NA | NA | NA |
| Nygaard et al, Clin Pharm Ther 2004 | Hepatotoxicity | (correlation) | NA | NA | NA |
| Odahara et al, PLoS One 2015 | Toxicity in general | Leukopenia (as below), hepatitis (ALT and AST over ULN), alopecia, agranulocytosis, rash. | 450 | NA | NA |
|  | Leukopenia | WBC < 2.5 x 10^9^/L | 450 | NA | NA |
| Ohtsuka et al, J Gastroenterol Hepatol 2010 | Leukopenia | (correlation) | NA | NA | NA |
| Ooi et al, Aliment Pharm Ther 2007 | Leukopenia | WBC < 4 x 10^9^/L | NA | NA | NA |
| Pranzatelli et al, J Clin Exp Immunol 2017 | Leukopenia | (correlation) | NA | NA | NA |
| Rae et al, J Neuroimmunol 2016 | Lymphopenia | (correlation) | NA | NA | NA |
| Sandborn et al, Gastroenterology 1999 | Leukopenia | (correlation) | NA | NA | NA |
| Schmiegelow et al, Cancer Chemother Pharmacol 1990 | Leukopenia | (correlation) | NA | NA | NA |
| Shaye et al, Am J Gastroenterol 2007 | Hepatotoxicity | ALT or AST > 2 x ULN or total bilirubin > 2 mg/dL | NA | 5300 | NA |
| Stoneham et al, Br J Haematol 2003 | Veno-occlusive disorder | At least 2 of the following: tender hepatomegaly, ascites, hyperbilirubinemia (> 17 µmol/L) or thrombocytopenia (platelet count < 75 x 10^9^/L) in the absence of neutropenia, of at least 4 weeks’ duration. | NA | NA | NA |
| Thomas et al, Inflamm Bowel Dis 2003 | Anaemia | (correlation) | NA | NA | NA |
|  | Leukopenia | (correlation) | NA | NA | NA |
|  | Thrombocytopenia | (correlation) | NA | NA | NA |
| Wojtuskiewicz et al, Nucleos Nucleot Nucl 2014 | Leukopenia | WBC < 2 x 10^9^/L | NA | NA | NA |
| Wong et al, Aliment Pharmacol Ther 2016 | Hepatotoxicity | ALT > 2 x ULN or ALT/ALP ratio ≥ 5 | NA | 3615 | NA |
| Wong et al, J Crohn Colitis 2017 | Leukopenia | WBC < 3 x 10^9^/L | 213 | 3525 | NA |
| Wright et al, Gut 2004 | Hepatotoxicity | ALT > ULN | NA | NA | NA |
| Yarur et al, J Clin Gastroenterol 2018 | Leukopenia | WBC < 3 x 10^9^/L | NA | NA | NA |
| Zochowska et al, Transplant Proc 2016 | Anaemia | (correlation) | NA | NA | NA |
|  | Leukopenia | (correlation) | NA | NA | NA |
|  | Thrombocytopenia | (correlation) | NA | NA | NA |
|  | Hepatotoxicity | (correlation) | NA | NA | NA |
|  | Hepatotoxicity | ALT and/or AST ≥ 1.5 x ULN | NA | 5700 | 20 |

ALP: alkaline phosphatase; ALT: alanine aminotransferase; ANC: absolute neutrophil count; AST: aspartate aminotransferase; GGT: gamma-glutamyl transpeptidase; Hb: haemoglobin; NA: non-applicable; ULN: upper limit of normal; WBC: white blood cell count

* after conversion to values comparable to Lennard method (converted values in italic), except when measurement method wasn’t specified

** all values in pmol/8x10^8^ red blood cells
