# Supplementary material for: Thiopurines’ Metabolites and Drug Toxicity: A Meta-Analysis
Source: J Clin Med. 2020 Jul 13;9(7):2216. doi: 10.3390/jcm9072216 (PMC7408995; doi:10.3390/jcm9072216)
Supplement: Supplementary file 1 [file jcm-09-02216-s001.zip › Table S2.docx]

**Supplementary Table 2: Measures of performance of different 6-TGN cut-offs for leukopenia occurrence** (calculated with the method described *in Steinhauser S, Schumacher M, Rucker G. Modelling multiple thresholds in meta-analysis of diagnostic test accuracy studies. BMC medical research methodology. 2016;16(1):97)*

| **6-TGN cut-off**  **(pmol/8×108 RBC)** | **Sensitivity** | **95% Confidence interval** | **Specificity** | **95% Confidence interval** |
| --- | --- | --- | --- | --- |
| 58 | **0.950** | **0.801-0.989** | 0.182 | 0.012-0.0804 |
| 100 | 0.856 | 0.634-0.953 | 0.347 | 0.056-0.825 |
| **135** | **0.754** | **0.479-0.911** | **0.464** | **0.126-0.839** |
| 150 | 0.710 | 0.418-0.893 | 0.506 | 0.162-0.845 |
| 200 | 0.566 | 0.251-0.836 | 0.621 | 0.299-0.863 |
| 250 | 0.445 | 0.149-0.787 | 0.702 | 0.430-0.880 |
| 300 | 0.350 | 0.091-0.744 | 0.518 | 0.534-0.897 |
| 350 | 0.278 | 0.058-0.707 | 0.803 | 0.609-0.914 |
| 904 | 0.046 | 0.003-0.471 | **0.950** | **0.797-0.989** |

Best sensitivity, specificity and optimal cut-off level are shown in bold.
