# Supplementary figures and images for: Thiopurines’ Metabolites and Drug Toxicity: A Meta-Analysis
Source: J Clin Med. 2020 Jul 13;9(7):2216. doi: 10.3390/jcm9072216 (PMC7408995; doi:10.3390/jcm9072216)

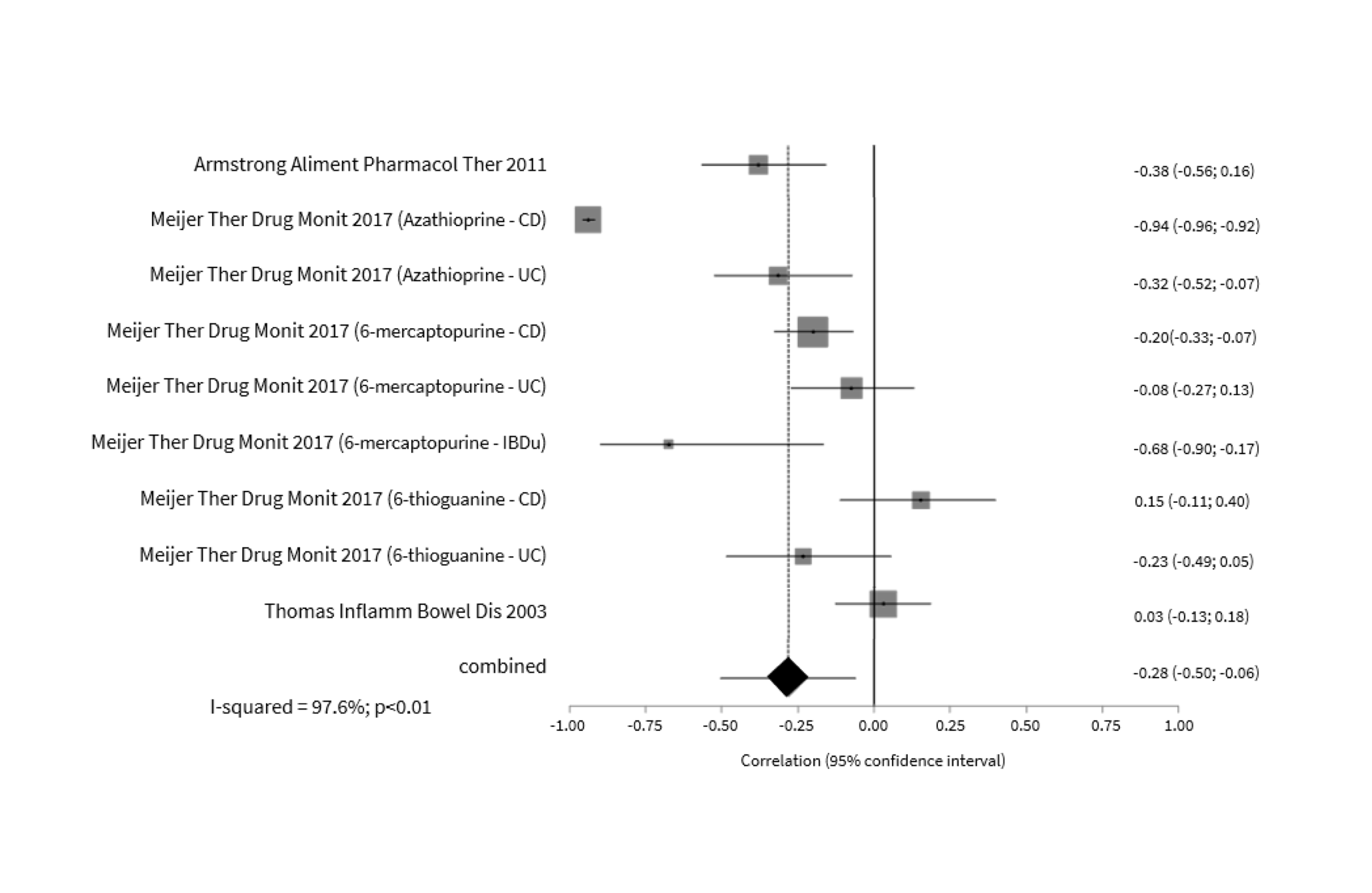

Supplement: Supplementary file 1 [file jcm-09-02216-s001.zip › Figure S4.tiff]

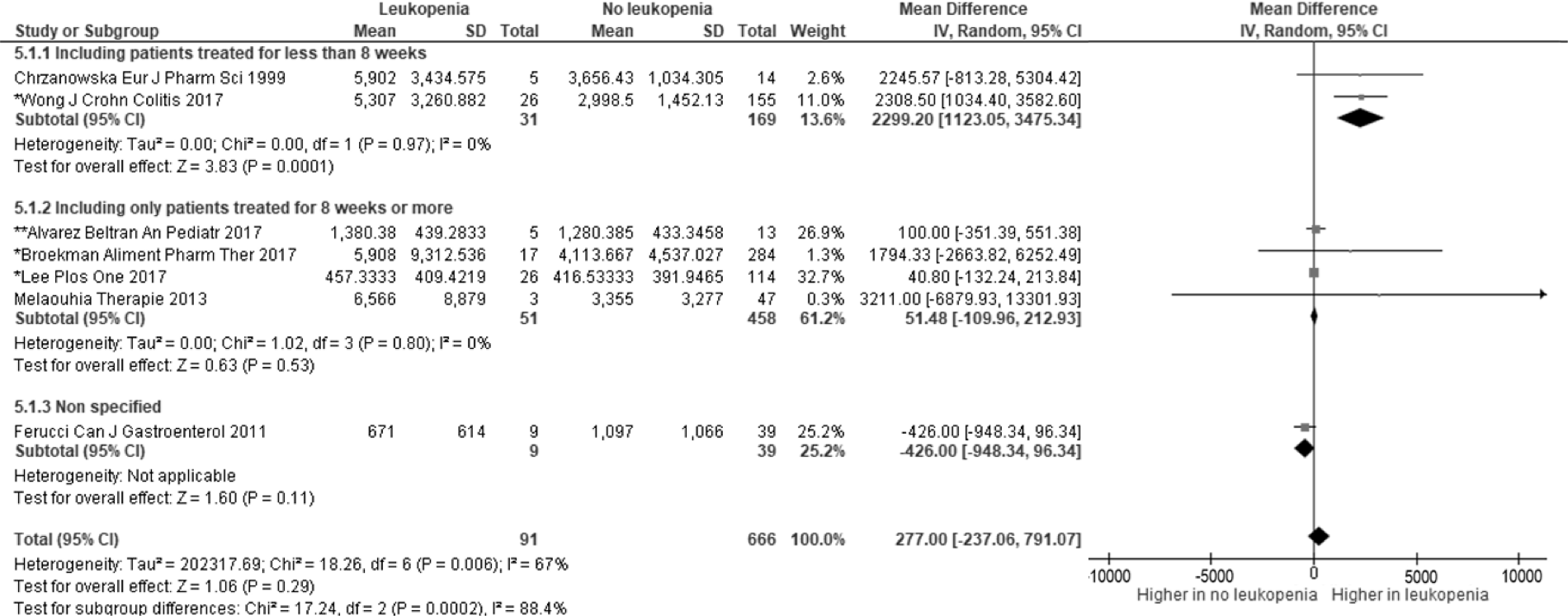

Supplement: Supplementary file 1 [file jcm-09-02216-s001.zip › Figure S5b.tif]

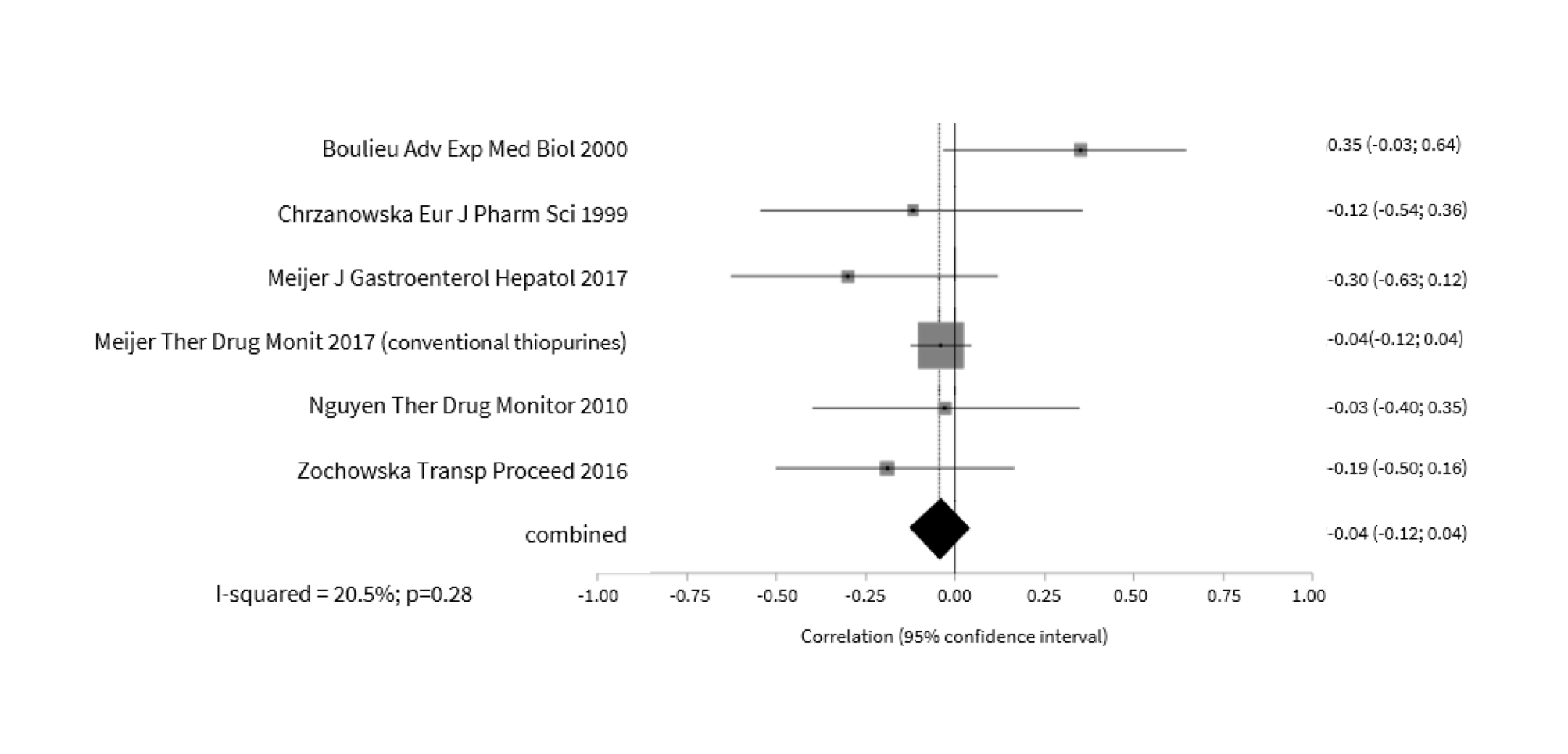

Supplement: Supplementary file 1 [file jcm-09-02216-s001.zip › Figure S6.tiff]

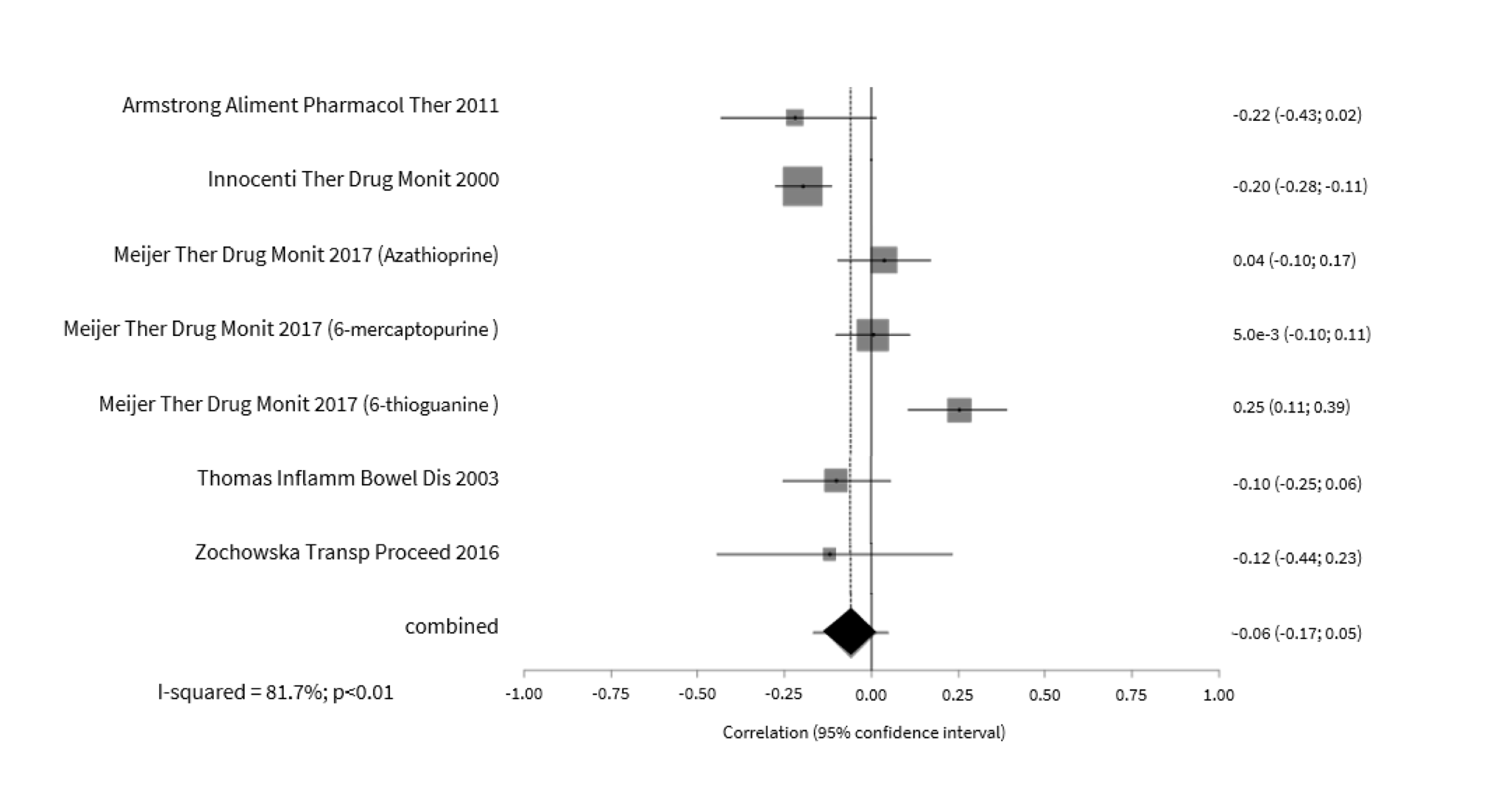

Supplement: Supplementary file 1 [file jcm-09-02216-s001.zip › Figure S9.tiff]
